# UHPLC-TQ-MS Coupled with Multivariate Statistical Analysis to Characterize Nucleosides, Nucleobases and Amino Acids in Angelicae Sinensis Radix Obtained by Different Drying Methods

**DOI:** 10.3390/molecules22060918

**Published:** 2017-06-01

**Authors:** Shaoqing Zhu, Sheng Guo, Jin-ao Duan, Dawei Qian, Hui Yan, Xiuxiu Sha, Zhenhua Zhu

**Affiliations:** Jiangsu Collaborative Innovation Center of Chinese Medicinal Resources Industrialization, and National and Local Collaborative Engineering Center of Chinese Medicinal Resources Industrialization and Formulae Innovative Medicine, Nanjing University of Chinese Medicine, Nanjing 210023, China; zhushaoqing1505@163.com (S.Z.); qiandw@njucm.edu.cn (D.Q.); glory-yan@163.com (H.Y.); shaxiu901128@yeah.net (X.S.); 04040416@163.com (Z.Z.)

**Keywords:** *Angelica sinensis*, nucleosides, nucleobases, amino acids, UHPLC-TQ-MS

## Abstract

To explore the nutrients in roots of *Angelica sinensis* (Angelicae Sinensis Radix, ASR), a medicinal and edible plant, and evaluate its nutritional value, a rapid and reliable UHPLC-TQ-MS method was established and used to determine the potential nutritional compounds, including nucleosides, nucleobases and amino acids, in 50 batches of ASR samples obtained using two drying methods. The results showed that ASR is a healthy food rich in nucleosides, nucleobases and amino acids, especially arginine. The total average content of nucleosides and nucleobases in all ASR samples was 3.94 mg/g, while that of amino acids reached as high as 61.79 mg/g. Principle component analysis showed that chemical profile differences exist between the two groups of ASR samples prepared using different drying methods, and the contents of nutritional compounds in samples dried with the tempering-intermittent drying processing method (TIDM) were generally higher than those dried using the traditional solar processing method. The above results suggest that ASR should be considered an ideal healthy food and TIDM could be a suitable drying method for ASR when taking nucleosides, nucleobases and amino acids as the major consideration for their known human health benefits.

## 1. Introduction

*Angelica sinensis* (Oliv.) Diels, an Umbelliferous plant, is indigenous to China and widely cultivated in Gansu, Yunnan, Sichuan and Hubei provinces of China. The dried roots of *A. sinensis* (Angelicae Sinensis Radix, ASR), called Danggui in China, have been commonly used for thousands of years in Asia as a traditional Chinese medicine for replenishing and invigorating blood, regulating menstruation, relieving pain, and moistening the intestines [1], and also consumed as a dietary supplement for health care in Europe and America. Phytochemical studies have revealed that ASR contains various constituents, including phenolic acids and their esters [2,3], phthalides [4], polysaccharides [5], inorganic elements [6], etc. Pharmacological studies have shown that these components from ASR exhibit multiple bioactivities, such as inhibition of platelet aggregation, improvement of microcirculation, reduction of hypercholesterolemia and hyperglycemia, hepatic protective effect, prevention of gastric mucosal damage, which contribute much to the therapeutic effects of ASR [7,8,9].

In addition, there are reports that nucleosides, nucleobases and amino acids with great nutritional value, such as adenosine, arginine, γ-aminobutyric acid, tryptophan, and lysine, are found in ASR. Nucleosides and amino acids participate in the synthesis of DNA and proteins and many of them exhibit multiple bioactivities like antiplatelet aggregation [10], antiarrhythmic [11], antioxidant [12], and antiviral actions [13]. These compounds have been developed into medical or functional food products. Adenosine, a nucleoside in ASR, is used as an antiarrhythmic agent [11] to treat a number of supraventricular tachycardia that do not improve with vagal maneuvers [14]. Arginine, a conditionally essential amino acid for humans, plays an important role in cell division, healing of wounds, removing ammonia from the body, immune function, and the release of hormones [15,16]. Considering the benefit of these compounds for human health, several analytical methods to detect them have been reported to assess the quality of food or medicinal materials, such as fruits of *Ziziphus jujuba* [17,18], Astragali Radix [19], Notoginseng Radix et Rhizoma [20], pollen of *Typha angustifolia* [21], etc.

Since the ASR is increasingly being used in healthcare products with its human health benefits, it is necessary to develop a fast, convenient and effective method to clearly characterize and quantify the potential nutritional constituents such as nucleosides, nucleobases and amino acids. In addition, it was reported that the drying process used is an important factor for the formation of exterior and internal quality of herbal medicines, although its effect on these nutritional compounds in ASR has been scarcely reported, which has restricted the selection of a suitable drying method for ASR. For the above reasons, in the present study, a UHPLC-TQ-MS method to simultaneously detect and quantitate nucleosides, nucleobases and amino acids in ASR was developed and validated. The method was then applied to analyze 50 samples of ASR and to evaluate the effect of different drying methods on these compounds in ASR.

## 2. Results and Discussion

### 2.1. Optimization of the Extraction Procedure

The extraction conditions used were, with some modifications, from a literature report, [17], where ultrasonic extraction with water was shown to be more efficient than refluxing and other solvents. The other extraction variables, including solvent volume (25 and 50 mL) and extraction time (15, 30, 45 and 60 min), were investigated on sample 1. While one of the parameters was being determined, the others were set at their default values. The results revealed that after the sample was extracted for 30 min, the total contents of the 40 analytes did not increase with further extension of the extraction time, and in some cases, the contents of some analytes even decreased because the long duration of the sonication led to the degradation of the more unstable components.

### 2.2. Optimization of MS Conditions

To select a proper transition for the MS/MS detection of the analytes, all the compounds were examined separately in direct infusion mode by the full-scan MS method in both positive and negative ionization modes. It was observed that all the standards presented higher sensitivity and clearer mass spectra in positive ion mode than in negative ion mode, except for adenosine-5′-monophosphate (A-5′-P) and guanosine-5′-monophosphate (G-5′-P) which exhibited a high sensitivity in negative ion mode but no response in positive ion mode. Thus, both the positive and negative scanning modes were applied in the analysis. Next, multiple reaction monitoring (MRM) was applied. To improve the selectivity and sensitivity in the analysis, at least two precursor/product ion pairs were selected for detection of the amino acids and the most selective and specific transition was chosen for MRM determination. As a result, target peaks [M + H]^+^ were selected as the precursor ions for amino acids, and most of them had abundant product ions [M + H−46]^+^, corresponding to the neutral loss of formic acid by a rearrangement from [R-CH(NH_3_)-COOH]^+^ to [R-CH=NH_2_]^+^, which is a specific transition for most *α*-amino acids. In addition, product ions [M + H−63]^+^ were also commonly observed in amino acids, which were consistent with [M + H-HCOOH-NH_3_]^+^. These were the most abundant transitions for glutamine, lysine and ornithine. For γ-aminobutyric acid, [M + H−17]^+^ and [M + H−35]^+^ were obtained. The former, corresponding to the loss of NH_3_, was selected as the quantitation MRM transition with greater abundance. For nucleosides and nucleobases, [M + H]^+^ target peaks were selected as the precursor ions in positive mode, except for A-5′-P and G-5′-P which as mentioned, were detected in negative mode with [M − H]^−^. As for MRM transitions, [M + H-deoxyribose]^+^ and [M + H-ribose]^+^ were selected as product ions for most of the nucleosides. For the nucleobases, including thymine, adenine, hypoxanthine, uracil and guanine, we had tried to obtain intensive product ions by optimization of the MS parameters, especially the collision energy. However, the abundance of product ions for these analytes were too low for MRM detection, which was consistent with a previous report [22]. Thus, single ion monitoring (SIM) was used for the quantitative determination of these five nucleobases. All the MRM/SIM transitions and parameters applied in the study are listed in Table 1.

### 2.3. Investigation of the UHPLC Chromatographic Conditions

**Figure 1:**
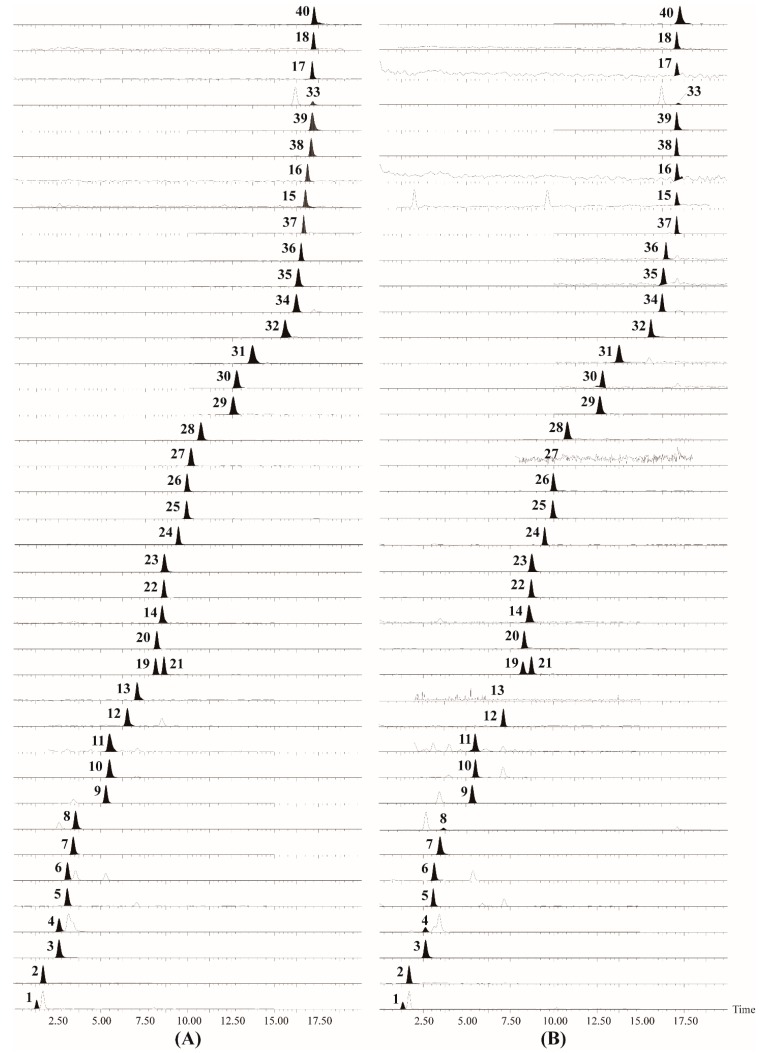
UPLC-TQ-MS chromatograms of mixed standards (**A**) and sample (**B**) for the 40 analytes in this study. The analytes numbers **1**–**40** are consistent with those in Table 1.

To obtain a desirable chromatographic profile with optimized retention time and peak shapes without obvious peak tailing, several factors were considered for optimization of the chromatographic conditions with the established MS/MS methods, such as column type, mobile phase composition and pH. Furthermore, two types of column, an Acquity BEH C_18_ (100 mm × 2.1 mm, 1.7 µm) column (Waters, Milford, MA, USA) and an Acquity BEH Amide (100 mm × 2.1 mm, 1.7 µm) column (Waters), were compared to optimize the retention times of the target analytes. The results showed that the latter gave a satisfactory retention and degree of separation for the 40 compounds. In addition, the Acquity BEH Amide column actually presented excellent suitability for highly polar compounds due to the strong hydrophilic interaction with the hydrophilic polar stationary phase [22,23], thus it was chosen for sample analysis. As for the mobile phase, the organic solvents methanol and acetonitrile were compared and the latter gave narrower peaks in a short analysis time, so acetonitrile was selected as the organic solvent. To get improved target peak shapes, mobile phase additives were also optimized. As reported in [24,25,26], the addition of ammonium acetate and ammonium formate to the mobile phase turned was helpful for improving the separation of nucleosides, their bases and amino acids. Thus, different concentrations of ammonium acetate, ammonium formate and their combination in the mobile phase were compared. The results showed that better peak shapes were observed when ammonium acetate and ammonium formate were mixed (1:1) rather than when either single was added, and narrower peaks were obtained when the buffer salt concentration increased. However, as the salt concentration increased, the intensity of the target peaks tended to decrease due to the ion suppression effect. Another disadvantage of high salt concentration lays in the poor solubility of the analytes in the organic phase. Based on the above considerations, the optimized buffer salt concentration was selected as 5 mM ammonium acetate and ammonium formate in aqueous solution and 1 mM of both in acetonitrile. As most of the analytes showed higher intensity [M + H]^+^ peaks in positive ionization mode, an acidic pH might help increase the sensitivity. Hence, different concentrations of formic acid (0.05%, 0.1%, 0.2%) in the above salt-containing mobile phase were investigated. The results showed that 0.2% formic acid (pH 2.5) mixed in the salt mobile phase afforded better peak shapes for most of the amino acids, except for some basic ones, such as histidine, arginine, and lysine, while gave better sensitivity and provided lower retention times than solutions with 0.05% and 0.1% formic acid. As a result, a mixed solution of aqueous solution (5 mM ammonium acetate and ammonium formate, 0.2% formic acid) and acetonitrile solution (1 mM ammonium acetate and ammonium formate, 0.2% formic acid) was chosen as the preferred mobile phase. Nonetheless, some analytes possess similar MS/MS characteristics, and must be separated well to avoid interference from each other. Such compounds include thymine (**1**) and thymidine (**2**), 2’-deoxyadenosine (**3**), adenine (**4**), and adenosine (**7**), guanine (**12**) and guanosine (**14**), uracil (**11**) and uridine (**5**), hypoxanthine (**6**), 2’-deoxyinosine (**8**), and inosine (**9**), Leu (**19**) and Ile (**21**), Glu (**32**), Gln (**34**), and Lys (**33**). To allow the accurate quantitative analysis of these groups of compounds with mutual interference, gradient elution was applied. The flow rate was set at 0.4 mL/min while the injection volume was set at 1 µL, and the column temperature was kept at 30 °C. The representative chromatograms of the 40 analytes are shown in Figure 1.

As observed in Figure 1, all the above mentioned interfering compound groups could be eluted with rather intense and narrow peaks with different retention times except for the compounds 2’-deoxyadenosine (**3**) and adenine (**4**), whose retention time was 2.6 min for both. In another words, adenine might be inaccurately quantified if it not separated successfully under the specific chromatographic condition in this experiment, because the *m*/*z* 136.0 peak in SIM could detect both compounds. In this case, we have tried our best to adjust the gradient elution to seek a better resolution of these components, but unfortunately, the results showed that the retention times of the two analytes were so close that a quantitative error could not be avoided in the analysis of adenine.

### 2.4. Method Validation

The proposed UHPLC method was validated by determining the linearity, LOD, LOQ, precision, stability, and recovery. The calibration curves of all compounds **1**–**40** exhibited excellent linear regressions with determination coefficients (r^2^) of more than 0.99 for each compound (Table 2). The LOD and LOQ were estimated to be 0.0001–0.1625 μg/mL and 0.0004–0.5412 μg/mL, respectively. The analytical precisions for a mixed standard solution of 40 compounds were acceptable with a RSD < 3.45% for intraday and RSD < 4.12% for interday. Stability RSDs of 40 analytes were under 5.02%, indicating the sample solutions were stable for at least two days when stored at 4 °C. Recovery tests showed that the method was accurate enough for the simultaneous determination of 40 nucleosides, their bases and amino acids in ASR with overall recoveries between 95.2–105.7%, for which the which RSDs < 3.80%. All the data is listed in Table 2. 

### 2.5. Identification and Quantification of the Nucleosides, Nucleobases and Amino Acids in the Samples

To date, a large number of studies have been conducted on the identification of markers for the related species [27,28], geographical origin [3,29,30], cultivation [3], harvest season [31,32], medicinal parts [3,33], drying method [31,34] and processing [27] of *A. sinensis*. However, most of them were focused on phenolic acids and their esters, phthalides, and polysaccharides, and few studies have concentrated on the characteristics of nucleosides, nucleobases and amino acids, which have great nutritional value. Thus, the established UHPLC-TQ-MS method was applied to identify these potential nutritional compounds and quantify them in 50 batches of ASR obtained by the primary drying processing method (PDPM) and the tempering-intermittent drying method (TIDM) (Table 3). The results showed (Tables S1 and S2) that almost all of the ASR samples were rich in the nucleosides, nucleobases and amino acids. All the compounds were found in ASR except guanine (**12**) and taurine (**27**); thymine (**1**), thymidine (**2**), 2′-deoxyinosine (**8**), and *trans*-4-hydroxy-l-proline (**30**) were also scarcely detected in the samples.

**Figure 2:**
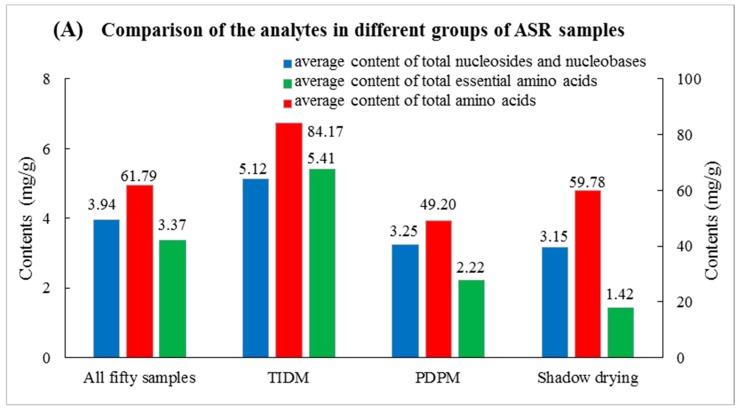

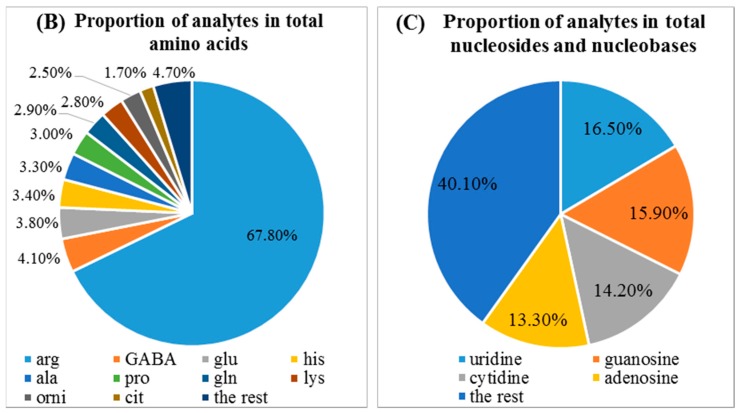
Comparison of nutrients in different ASR samples and proportion of the relatively abundant analytes in total amino acids, as well as in total nucleosides and nucleobases. (**A**) Comparison of the analytes in different groups of ASR samples; (**B**) Proportion of analytes in total amino acids; (**C**) Proportion of analytes in total nucleosides and nucleobases.

In order to clearly describe the overall composition and proportion of the various components in ASR, as well as the differences between the samples obtained using different drying methods, total amino acids, total essential amino acids, and total nucleosides and nucleobases, along with proportion of the most abundant and specific compounds were calculated and the results are shown in Figure 2. As observed in Figure 2A, the total contents of nucleosides and nucleobases in all ASR samples were 3.94 mg/g on average, while that of amino acids reached as high as 61.79 mg/g, among which total contents of essential amino acids were 3.37 mg/g, confirming the high nutritional value of ASR. As specifically abundant analytes, shown in Figure 2B,C, the mean contents of uridine, guanosine, cytidine and adenosine accounted for 16.5%, 15.9%, 14.2%, and 13.3% of the total nucleosides and nucleobases. As for amino acids, the mean content of arginine accounted for 67.8% of the total amino acids, much higher than other relatively abundant ones, such as γ-aminobutyric acid (4.1%), glutamic acid (3.8%), histidine (3.4%), alanine (3.3%), proline (3.0%), glutamine (2.9%), lysine (2.8%), ornithine (2.5%) and citrulline (1.7%). In plant physiology, studies [35] have shown that arginine is more important for plants than other nitrogenous nutrients because it participates in plant growth & development and resistance to stress through its polyamine (PA) and nitric oxide (NO) metabolites and the fact that the arginine content remains at higher levels in roots during the over-wintering period may explain the richness of arginine in ASR which grows in high altitude regions with cool or cold weather and is harvested between November and December. Considering the high content of arginine in ASR, along with the richness in the other basic nutrients of great benefits for humans, ASR may be developed as a supplementation product to maintain a normalized body.

As for the differences between samples obtained by different drying methods, it was found that the contents of most nutritional compounds in TIDM samples were higher than those in PDPM samples, as shown in Figure 2A. For example, the average contents of total amino acids and total nucleosides and nucleobases in the samples 1–18 (which were all obtained using the TIDM method) were 84.2 and 5.1 mg/g, respectively, while they were only 49.2 and 3.3 mg/g in PDPM samples (samples 19–29, 31–50), respectively. This result indicated that TIDM would be better choice for recovering of these compounds than PMPD and in this case, TIDM was more suitable for the drying processing in ASR cultivation regions.

For the samples obtained with shadow drying, only total amino acids reached the average level of all fifty samples, however, the contents of total nucleosides and nucleobases and total essential amino acids were both lower than the average of those obtained with TIDM or PDPM. The result indicated that it would not be appropriate to select shadow drying for the sake of retaining the nucleosides and nucleobases, as well as the essential amino acids. For the specific example of arginine, a predominant amino acid in many samples, its highest content reached 85.30 mg/g in sample 2 (a TIDM sample), whereas it was only 14.36 mg/g in sample 37 (a PDPM sample), as shown in Table S2. This phenomenon maybe due to the higher temperatures (≥40 °C) used in the TIDM process which can promote the degradation of macromolecular proteins and ribonucleic acids into smaller ones thus making them easier to extract into aqueous solutions. Another explanation could be that during the early stages of the drying process or drought stress under low temperatures (<30 °C) for a long time in the PDPM process, the root cells were still alive with strong respiration, metabolism, consumption of energy and organic matters, and that may lead to the poorer accumulation of nutritious components. Regardless of the reason, it is reasonable to conclude that the programmed tempering-intermittent drying method would be a better choice for drying ASR compared to the solar drying method which was widely used today.

### 2.6. Principal Component Analysis of the Samples

**Figure 3:**
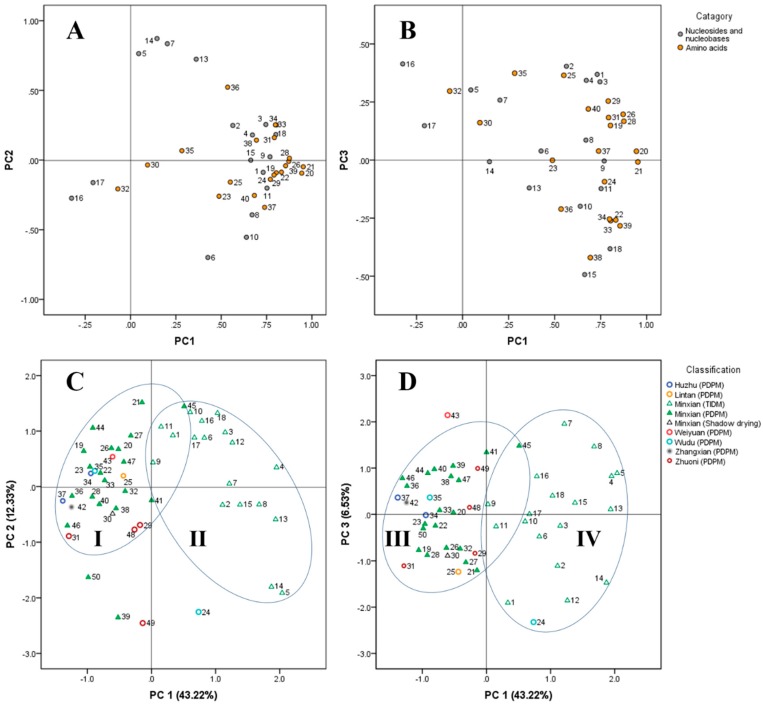
Loading plots and scatter plots obtained by PCA. (**A**) Loadings on PC 1 and PC 2 of all the analytes except for guanine and taurine; (**B**) Loadings on PC 1 and PC 3 of all the analytes except for guanine and taurine; (**C**) Scatter plots of the 50 ASR samples; (**D**) Scatter plots of the 50 ASR samples.

To evaluate the variation of ASR samples, especially that between the samples obtained using the TIDM and PDPM methods, a principal component analysis (PCA) was performed on the basis of the contents of all tested compounds. The first three principal components (PC 1, PC 2 and PC 3) with >62% of the whole variance were extracted for analysis. Among them, PC 1, PC 2 and PC 3 accounted for 43.22%, 12.33%, and 6.53% of the total variance, respectively. The remaining principal components that had minor effects on the model were discarded. The components loading matrix is shown in Figure 3A,B. According to the loadings, PC 1 had a good correlation with all the analytes, except for compounds **5**, **7**, **13**, **14**, **16**, **17**, **30**, **32**, and **35**. Of these excluded nine compounds, the former four exhibited good correlations with PC 2, but the latter five showed little correlations with either PC 2 or PC 3. In spite of this, the two main groups of ASR samples were separated to a large degree on PC 1 in the sample scatter plot, as shown in Figure 3C,D, where each sample was represented as a marker. As shown in Figure 3C,D, ASR samples from Minxian obtained with PDPM were almost separated from those with TIDM on PC 1 and PC 2 in two domains, namely **I** and **II**, and the two groups were also set apart on PC 1 and PC 3 in another two similar domains, namely **III** and **IV**. These observations suggested that there was significant difference between samples obtained with TIDM and PDPM in the profiles of the nucleosides, nucleobases and amino acids, which is also consistent with our previous results that the contents of most compounds determined in the TIDM samples were higher than those in PDPM samples and the PCA method could be used for discrimination between them. In addition, it was also noticeable that domain **I** and **II** along with **III** and **IV** overlapped each other, and it was reasonable to conclude that those samples being close to domain **I** and **III**, such as 9 and 11, were more similar in their chemical profiles to PDPM samples than other TIDM samples. As for the other samples from Gansu (including Lintan, Zhuoni, Weiyuan, and Wudu) and Qinghai (Huzhu) province, most of them shared the same domains, namely **I** or **III**, as samples from Minxian. Actually, they are all geographically close to each other and were dried with a unified primary drying method. As for sample 39 (Minxian, PDPM), it was excluded from the domain **I** on PC 2, suggesting a difference in the profiles of those compounds having good correlation with PC 2, which was confirmed by the lower contents of compounds **5**, **7**, **13**, **14** and **36** in sample 39 than in other samples in domain **I**. This result may be due to the specific process of decrustation used for the sake of exterior quality of sample 39 during the primary drying process. Samples 49, 50 and 24 shared similar chemical properties on PC 2 but were unfortunately separated from domain **I** which could be due to an uncontrolled pretreatment, and a lack of standard operation procedure (SOP) during the primary drying process.

## 3. Experimental Section

### 3.1. Chemicals, Reagents and Materials

Acetonitrile, methanol and formic acid were of HPLC grade (Merck, Darmstadt, Germany) and deionized water (H_2_O) was purified by a superpurification system (Eped Technology Development, Nanjing, China). Ammonium formate (Lingfeng Chemical Reagent, Shanghai, China) and ammonium acetate (Sinopharm Chemical Reagent, Shanghai, China) were both of analytical grade. Chemical standards of thymine (**1**), thymidine (**2**), 2′-deoxyadenosine (**3**), adenine (**4**), uridine (**5**), hypoxanthine (**6**), adenosine (**7**), inosine (**9**), cytosine (**10**), cytidine (**13**), guanosine (**14**), 2′-deoxyadenosine-5′-monophosphate (**15**), adenosine-5′-monophosphate (**16**), guanosine-5′-monophosphate (**17**), cytidine-5′-monophosphate (**18**), γ-aminobutyric acid (**23**), taurine (**27**), *trans*-4-hydroxy-l-proline (**30**), glutamine (**34**), asparagine (**36**), citrulline (**37**) and ornithine (**40**) were obtained from Sigma (St. Louis, MO, USA). Reference compounds of 2′-deoxyinosine (**8**) and uracil (**11**) were obtained from Aladdin Reagent Co. Ltd. (Shanghai, China). Chemical standards of guanine (**12**), leucine (**19**), phenylalanine (**20**), iso-leucine (**21**), tryptophan (**22**), methionine (**24**), l-proline (**25**), valine (**26**), tyrosine (**28**), alanine (**29**), threonine (**31**), glutamic acid (**32**), lysine (**33**), serine (**35**), arginine (**38**), and l-histidine (**39**) were obtained from Huixing Biochemical Reagent Ltd. (Shanghai, China). The purity of each compound was >98%, as determined by HPLC analysis. 

Fresh ASR (100 kg in total) was collected from Minxian, Gansu Province (China) in December 2014, and randomly divided into 19 batches, of which, 18 batches were placed in a drying oven, and dried by programmed the tempering-intermittent drying process (TIDM) with different series of parameters. For example, the series of parameters of 40 °C-12 h-60 °C means that a sample was dried at 40 °C to the point of moisture content being about 40%, and put out for 12-h tempering, then dried at 60 °C to the end when the moisture content less than 12%. All these parameters were selected from an optimization scheme. The other batch of ASR was obtained using shadow drying method performed in the laboratory. Specifically, it was dried in a cool and dry place at approximately 20 °C without direct sunlight and external heat source for about a month. In addition, 31 batches of ASR were collected from Gansu and Qinghai provinces (China) in December 2014, and they all were processed in the respective cultivation regions using the primary drying processing method (PDPM) including solar drying accompanied with some interventions such as decrustation, piling, and kneading, etc. All the information of these samples is listed in Table 3. Their botanical origin was confirmed as root of *Angelica sinensis* (Oliv.) Diels. by the corresponding author.

### 3.2. Apparatus and Chromatographic Conditions

Analysis was carried out on an Acquity UHPLC system (Waters, Milford, MA, USA) consisting of a quaternary pump solvent management system, an online degasser, an autosampler, and a Xevo™ Triple Quadrupole MS (Waters) equipped with an electrospray ionization (ESI) source. Chromatographic analysis was performed on an Acquity UPLC BEH Amide (100 mm × 2.1 mm, 1.7 µm) column with a column temperature at 30 °C. The raw data was acquired and processed with Masslynx 4.1 software. The mobile phase was composed of A (5 mM ammonium formate and ammonium acetate, 0.2% formic acid) and B (acetonitrile with 1 mM ammonium formate, ammonium acetate, and 0.2% formic acid) with a gradient elution: 1–3 min, 10% A; 3–9 min, 10–18% A; 9–15 min, 18–20% A; 15–16 min, 20–46% A. The flow rate was 0.4 mL/min. The triple quadrupole (TQ) mass spectrometer was operated in both positive and negative ion mode with the same capillary voltage of 3 kV, a sampling cone voltage of 30 V, a cone gas flow of 20 L/h, a desolvation gas flow of 1000 L/h, a desolvation temperature of 350 °C, a source temperature of 120 °C, a collision energy of 6 V, and full-scan spectra from 100 to 1000 Da. High-purity nitrogen was used as the nebulizer and auxiliary gas and argon was utilized as the collision gas. 

### 3.3. Preparation of Standard Solution

A mixed standard stock solution containing the reference compounds **1**–**40** was prepared in methanol/water (9:1, *v*/*v*) at concentrations of 26.2, 30.8, 21.3, 22.6, 23.6, 23.6, 28.0, 25.4, 28.6, 27.0, 24.0, 23.8, 23.6, 24.8, 24.0, 22.4, 21.2, 24.0, 29.2, 29.4, 31.0, 25.2, 31.4, 24.4, 25.2, 37.2, 35.0, 37.6, 29.4, 35.4, 26.2, 31.4, 29.6, 32.0, 40.8, 35.2, 29.8, 28.0, 36.0, and 24.6 µg/mL, respectively. Working standard solutions for calibration curves were prepared by diluting the mixed standard solution with 10% methanol at different concentrations.

### 3.4. Preparation of Sample Solutions

A dried sample was pulverized and sieved (40 mesh). 1.0 g powder, accurately weighed, was sonicated (100 Hz, 25 °C) with 50 mL water for 30 min in a 100 mL glass-stoppered conical flask. Solvent was added after extraction if there was any weight loss. After centrifugation (13,000 r/min, 10 min) and filtration (0.22 µm membrane filter), the supernatant was stored (4 °C) at a sample plate before injection into the UHPLC system for analysis.

### 3.5. Method Validation

#### 3.5.1. Linearity and Sensitivity

The calibration curve was constructed by plotting the peak area against the corresponding concentration of each compound. The lowest concentration of working solution for calibration was further diluted with 10% methanol to determine the limit of detection (LOD) and the limit of quantitation (LOQ) at a signal to noise ratio (s/n) of 3:1 and 10:1, respectively.

#### 3.5.2. Precision and Stability

The intra- and inter-day precisions were evaluated by replicate injection of a standard solution containing 40 analytes for six times on the same day and another three consecutive days, respectively. The stability was evaluated by replicate injection of a sample solution every 4 h for two days.

#### 3.5.3. Accuracy

The recovery test was conducted to assess the accuracy. Determined in sextuplicate, the 40 standards (approximately 100% the content in sample) were added to an accurately weighed sample (0.5 g) which was subsequently extracted with the above-mentioned method and analyzed.

#### 3.5.4. Identification and Quantification

The identification of the nucleosides, nucleobases, and amino acids was achieved by comparing the retention time as well as the SIM or MRM transitions of target peaks with those of the standards by UHPLC-TQ-MS. Quantification was performed on the basis of linear calibration plots of the peak areas versus the concentration.

#### 3.5.5. Data Processing and Statistical Analysis

The UHPLC-TQ-MS were processed using the TargetLynx application manager for the quantification of analytes. Principle component analysis (PCA) was performed using SPSS 16.0 software. In this study, the contents of the 40 markers analyzed from the 50 samples composed a data matrix with 50 rows and 40 columns, which was used for PCA after normalization. The first three principal components (PCs) were extracted, and the scatter plot were obtained by plotting the scores of PC 1 versus PC 2 and PC 1 versus PC 3.

## 4. Conclusions

Within the aim of exploring the nutrients in ASR and evaluating its quality and safety, a rapid and reliable UHPLC-TQ-MS analysis was established to determine the potential nutritional compounds, including nucleosides, nucleobases and amino acids, in 50 batches of ASR samples obtained using two different drying methods. The analysis results showed that ASR is a healthy food rich in amino acids, especially arginine, as well as nucleosides and nucleobases. Two main groups of ASR samples were well classified by PCA according to different drying method, and the contents of the nutritional compounds in the samples obtained with TIDM were mostly higher than those obtained with PMPD, which suggests that TIDM could be a suitable drying method for ASR. However, the mechanism for this phenomenon was not clear and should be further elucidated.

## Figures and Tables

**Table 1:** Precusor/product ion pairs and parameters for SIM/MRM of compounds used in this study.

No.	Compound	*t*_R_ (min)	[M + H]^+^ (*m*/*z*)	[M − H]^−^ (*m*/*z*)	MRM Transitions/SIM	Cone Voltage (V)	Collision Energy (eV)
**Nucleosides and nucleobases:**							
**1**	Thymine	1.3	127.0		127.0	30	15
**2**	Thymidine	1.7	243.0		243.0→126.9	10	10
**3**	2′-Deoxyadenosine	2.6	252.0		252.0→135.9	16	14
**4**	Adenine	2.6	136.0		136.0	30	20
**5**	Uridine	3.1	245.0		245.0→112.9	10	10
**6**	Hypoxanthine	3.1	137.0		137.0	16	8
**7**	Adenosine	3.4	268.0		268.0→135.9	22	18
**8**	2′-Deoxyinosine	3.5	253.0		253.0→136.9	22	12
**9**	Inosine	5.3	269.0		269.0→136.9	10	14
**10**	Cytosine	5.5	112.1		112.1→94.9	32	18
**11**	Uracil	6.0	112.9		112.9	30	15
**12**	Guanine	6.5	152.0		152.0	30	20
**13**	Cytidine	7.0	244.0		244.0→111.9	28	10
**14**	Guanosine	8.4	284.1		284.1→152.0	14	14
**15**	2′-Deoxyadenosine-5′-monophosphate	16.8	332.0		332.0→135.9	20	16
**16**	Adenosine-5′-monophosphate	16.9		346.2	346.2→134.0	30	26
**17**	Guanosine-5′-monophosphate	17.2		362.2	362.2→211.0	26	16
**18**	Cytidine-5′-monophosphate	17.3	324.0		324.0→111.9	16	14
**Amino acids:**							
**19**	l-Leucine	8.0	132.1		132.1→86.1 ^a^	16	10
					132.1→69.1	16	18
**20**	l-Phenylalanine	8.3	166.1		166.1→120.1 ^a^	18	14
					166.1→ 103.0	18	22
**21**	iso-Leucine	8.6	132.1		132.1→86.1 ^a^	16	10
					132.1→69.1	16	18
**22**	l-Tryptophan	8.6	205.1		205.1→146.0 ^a^	16	18
					205.1→118.0	16	24
**23**	γ-Aminobutyric acid	8.7	103.9		103.9→87.0 ^a^	12	6
					103.9→68.9	16	14
**24**	l-Methionine	9.4	150.1		150.1→104.0 ^a^	14	10
					150.1→56.0	14	16
**25**	l-Proline	9.9	116.0		116.0→70.0 ^a^	20	10
					116.0→43.0	20	20
**26**	l-Valine	9.9	118.0		118.0→72.1 ^a^	12	10
					118.0→55.1	12	18
**27**	Taurine	10.1	126.0		126.0→44.0 ^a^	24	14
					126.0→108.0	10	10
**28**	l-Tyrosine	10.7	182.1		182.1→136.0 ^a^	16	16
					182.1→91.0	16	6
**29**	l-Alanine	12.5	90.0		90.0→44.0 ^a^	16	10
					90.0→62.0	16	6
**30**	*trans*-4-Hydroxy-l-proline	12.7	132.0		132.0→67.9 ^a^	18	16
					132.0→86.0	18	10
**31**	l-Threonine	13.6	120.0		120.0→74.0 ^a^	38	20
					120.0→93.0	38	14
**32**	l-Glutaminc acid	15.5	147.9		147.9→83.9 ^a^	12	14
					147.9→56.0	12	12
**33**	l-Lysine	17.2	147.0		147.0→83.9 ^a^	14	14
					147.0→56.1	14	24
**34**	l-Glutamine	16.2	147.0		147.0→83.9 ^a^	8	16
					147.0→56.0	8	24
**35**	l-Serine	16.3	106.0		106.0→60.0 ^a^	14	8
					106.0→70.0	14	14
**36**	l-Asparagine	16.5	132.9		132.9→73.9 ^a^	12	14
					132.9→87.0	12	18
**37**	l-Citrulline	16.7	176.0		176.0→69.9 ^a^	16	20
					176.0→106.0	16	10
**38**	l-Arginine	17.1	175.2		175.2→70.0 ^a^	22	18
					175.2→60.0	22	14
**39**	l-Histidine	17.2	156.1		156.1→110.0 ^a^	20	16
					156.1→83.0	20	20
**40**	l-Ornithine	17.3	133.0		133.0→69.9 ^a^	14	14
					133.0→ 116.1	12	12

^a^ Transition used for quantitation.

**Table 2:** Linear regression, LOD, LOQ and precision of 40 standard compounds and stability and recovery of the analytes in a sample solution.

No. ^b^	Regression Equation ^c^	r^2^	Linear Range (µg/mL)	LOD (µg/mL)	LOQ (µg/mL)	Precision (RSD, %)		Stability (RSD, %)	Recovery (*n* = 6)	
						Intraday (*n* = 6)	Interday (*n* = 18)		Mean, %	RSD, %
**1**	*y* = 392.6*x* − 49.916	0.9954	0.524–26.2	0.0586	0.1953	2.97	3.99	3.98	102.5	3.19
**2**	*y* = 630*x* + 57.152	0.9948	0.616–30.8	0.0177	0.0588	2.77	4.93	4.85	99.9	1.29
**3**	*y* = 180160*x* + 97682	0.9970	0.425–21.3	0.0001	0.0004	2.16	4.14	4.02	102.6	4.81
**4**	*y* = 11809*x* + 4671.1	0.9952	0.452–22.6	0.0018	0.0061	2.41	4.08	4.76	103.8	3.08
**5**	*y* = 363.6*x* + 120.5	0.9995	0.472–23.6	0.0611	0.2036	2.97	4.8	4.19	104.6	1.32
**6**	*y* = 3139.2*x* + 2525	0.9963	0.472–23.6	0.0068	0.0227	2.41	4.53	4.59	102.7	3.89
**7**	*y* = 141382*x* + 94037	0.9974	0.560–28.0	0.0002	0.0005	3.09	3.65	4.05	104.9	2.50
**8**	*y* = 3774.2*x* + 1124.3	0.9998	0.508–25.4	0.0059	0.0197	2.57	4.62	4.27	103.1	4.08
**9**	*y* = 4960.3*x* + 4375.9	0.9965	0.572–28.6	0.0045	0.0149	2.23	4.49	4.24	104.2	2.35
**10**	*y* = 5863.7*x* + 298.23	0.9999	0.540–27.0	0.0038	0.0128	3.15	3.87	3.81	104.4	3.95
**11**	*y* = 891.7*x* + 849.87	0.9991	0.480–24.0	0.0245	0.0817	3.09	4.09	4.17	103.5	3.74
**12**	*y* = 905.2*x* + 781.29	0.9991	0.476–23.8	0.0242	0.0807	3.92	4.2	4.44	97.8	1.20
**13**	*y* = 187.1*x* + 11.88	0.9933	0.472–23.6	0.1181	0.3933	3.45	4.12	4.81	96.4	4.59
**14**	*y* = 961.0*x* − 72.951	0.9948	0.496–24.8	0.0243	0.0809	2.34	4.17	4.28	95.2	2.78
**15**	*y* = 210.9*x* − 44.971	0.9980	0.480–24.0	0.1088	0.3623	2.89	3.09	3.24	102	3.11
**16**	*y* = 338*x* − 21.961	0.9968	0.447–22.4	0.0668	0.2224	3.02	3.45	3.66	98.6	2.82
**17**	*y* = 618*x* − 5.3866	0.9996	0.424–21.2	0.0364	0.1213	3.22	3.4	3.27	105.7	1.62
**18**	*y* = 735*x* − 23.523	0.9967	0.480–24.0	0.0315	0.1048	3.16	3.78	4.03	104.5	3.80
**19**	*y* = 3232.4*x* + 849.76	0.9992	0.584–29.2	0.0068	0.0227	3.23	4.85	4.33	97.7	3.16
**20**	*y* = 11236*x* − 5692	0.9967	0.588–29.4	0.0020	0.0067	2.18	4.34	4.68	99.8	1.01
**21**	*y* = 2574.4*x* + 2231.2	0.9994	0.620–31.0	0.0086	0.0285	3.2	3.75	3.61	97.5	4.05
**22**	*y* = 5387.7*x* − 845.81	0.9957	0.504–25.2	0.0041	0.0138	2.09	3.63	3.73	101.1	3.41
**23**	*y* = 135.8*x* + 173.86	0.9958	0.628–31.4	0.1607	0.5352	2.62	3.58	3.18	95.5	4.88
**24**	*y* = 402.8*x* + 122.7	0.9989	0.488–24.4	0.0559	0.186	2.31	3.87	3.94	98.9	2.49
**25**	*y* = 548.0*x* − 5.2532	0.9970	0.504–25.2	0.0419	0.1394	3.48	4.24	4.26	98.2	3.08
**26**	*y* = 283.7*x* + 154.16	0.9994	0.744–37.2	0.0789	0.2627	2.34	3.76	3.45	97.2	3.31
**27**	*y* = 860*x* − 3.1856	0.9992	0.700–35.0	0.0263	0.0875	2.76	3.65	3.45	100.5	2.62
**28**	*y* = 1042*x* + 83.479	0.9980	0.752–37.6	0.0212	0.0705	2.64	4.13	2.16	100.0	1.17
**29**	*y* =518*x* + 31.816	0.9980	0.588–29.4	0.04308	0.1435	2.81	4.42	4.38	99.3	4.94
**30**	*y* = 136*x* + 100.17	0.9999	0.708–35.4	0.1625	0.5412	3.23	4.87	4.91	103.2	1.69
**31**	*y* = 861*x* + 98.616	0.9962	0.524–26.2	0.0253	0.0843	2.59	3.9	3.55	98	3.40
**32**	*y* = 539*x* − 15.987	0.9973	0.628–31.4	0.0416	0.1385	2.59	3.33	3.75	97.5	2.71
**33**	*y* = 295.7*x* + 241.23	0.9972	0.592–29.6	0.0749	0.2494	2.38	3.63	3.52	102.5	2.08
**34**	*y* = 550.3*x* + 519.33	0.9901	0.640–32.0	0.0412	0.1373	2.66	4.79	4.40	105.4	2.92
**35**	*y* = 780*x* + 24.97	0.9984	0.816–40.8	0.0272	0.0905	2.75	4.78	4.18	97.4	2.88
**36**	*y* = 940*x* − 21.517	0.9999	0.704–35.2	0.0178	0.0592	2.9	4.39	4.83	96.2	3.80
**37**	*y* = 368*x* − 19.204	0.9985	0.596–29.8	0.0624	0.2079	2.79	4.71	4.73	97.7	3.56
**38**	*y* = 465*x* + 432.16	0.9979	0.560–28.0	0.0465	0.1548	2.94	3.89	3.96	98.5	4.06
**39**	*y* = 266.5*x* + 5.1122	1.000	0.720–36.0	0.0845	0.2813	2.15	3.12	3.27	96.5	3.13
**40**	*y* = 162*x* − 17.799	0.9999	0.492–24.6	0.1457	0.4852	2.99	3.24	3.41	105.0	4.00

^a^ The sample was prepared from No. 1; ^b^ The 40 analytes was the same as in Table 1; ^c^ *x* is the concentration of each compound in µg/mL; *y* is the peak area of the respective compound.

**Table 3:** Cultivation regions and drying methods of the 50 batches of samples.

Sample	Cultivation Regions	Drying Method	Sample	Cultivation Regions	Drying Method
1	Dazhai, Minxian, Gansu	40 °C-12 h-50 °C, TIDM ^a^	26	Zhangjiaping, Minxian, Gansu	PDPM
2	Dazhai, Minxian, Gansu	40 °C-24 h-60 °C, TIDM	27	Ningba, Minxian, Gansu	PDPM
3	Dazhai, Minxian, Gansu	40 °C-36 h-70 °C, TIDM	28	Jiaochangzhuang, Minxian, Gansu	PDPM
4	Dazhai, Minxian, Gansu	50 °C-24 h-60 °C, TIDM	29	Bailin, Zhuoni, Gansu	PDPM
5	Dazhai, Minxian, Gansu	50 °C-36 h-70 °C, TIDM	30	Dazhai, Minxian, Gansu	Shadow drying ^c^
6	Dazhai, Minxian, Gansu	50 °C-12 h-50 °C, TIDM	31	Shihuijiao, Zhuoni, Gansu	PDPM
7	Dazhai, Minxian, Gansu	60 °C-12 h-70 °C, TIDM	32	Jizhai, Minxian, Gansu	PDPM
8	Dazhai, Minxian, Gansu	60 °C-24 h-50 °C, TIDM	33	Sangjiagou, Minxian, Gansu	PDPM
9	Dazhai, Minxian, Gansu	60 °C-36 h-60 °C, TIDM	34	Zongzhai, Huzhu, Qinghai	PDPM
10	Dazhai, Minxian, Gansu	40 °C-36 h-60 °C, TIDM	35	Aihaoping, Wudu, Gansu	PDPM
11	Dazhai, Minxian, Gansu	40 °C-12 h-70 °C, TIDM	36	Caojiazhuang, Minxian, Gansu	PDPM
12	Dazhai, Minxian, Gansu	40 °C-24 h-50 °C, TIDM	37	Leijiabao, Huzhu, Qinghai	PDPM
13	Dazhai, Minxian, Gansu	50 °C-36 h-50 °C, TIDM	38	Dazhai, Minxian, Gansu	PDPM
14	Dazhai, Minxian, Gansu	50 °C-12 h-60 °C, TIDM	39	Nidizu, Minxian, Gansu	PDPM
15	Dazhai, Minxian, Gansu	50 °C-24 h-70 °C, TIDM	40	Jiangjia, Minxian, Gansu	PDPM
16	Dazhai, Minxian, Gansu	60 °C-24 h-70 °C, TIDM	41	Huigou, Minxian, Gansu	PDPM
17	Dazhai, Minxian, Gansu	60 °C-36 h-50 °C, TIDM	42	Caizichuan, Zhangxian, Gansu	PDPM
18	Dazhai, Minxian, Gansu	60 °C-12 h-60 °C, TIDM	43	Luojiamo, Weiyuan, Gansu	PDPM
19	Taizi, Minxian, Gansu	PDPM ^b^	44	Liujia, Minxian, Gansu	PDPM
20	Shendu, Minxian, Gansu	PDPM	45	Dalu, Minxian, Gansu	PDPM
21	Minxian, Dingxi, Gansu	PDPM	46	Lamei, Minxian, Gansu	PDPM
22	Qingshui,Minxian, Gansu	PDPM	47	Xiaohong, Minxian, Gansu	PDPM
23	Zhuoluo, Minxian, Gansu	PDPM	48	Lalu, Zhuoni, Gansu	PDPM
24	Fangping, Wudu, Gansu	PDPM	49	Shangzhuo, Zhuoni, Gansu	PDPM
25	Zongzhai, Lintan, Gansu	PDPM	50	Guoha, Minxian, Gansu	PDPM

^a^ TIDM represented programmed tempering-intermittent drying process method; ^b^ PDPM represented primary drying processing method; ^c^ Shadow drying represented the shadow drying process method by which samples were dried in a cool and dry place at approximately 20 °C without direct sunlight for about a month.

## Supplementary Materials

Supplementary materials are available online.
