# Supplementary material for: UHPLC-TQ-MS Coupled with Multivariate Statistical Analysis to Characterize Nucleosides, Nucleobases and Amino Acids in Angelicae Sinensis Radix Obtained by Different Drying Methods
Source: Molecules. 2017 Jun 1;22(6):918. doi: 10.3390/molecules22060918 (PMC6152706; doi:10.3390/molecules22060918)
Supplement: Supplementary file 1 [file molecules-22-00918-s001.pdf]

Supporting Information

UHPLC-TQ-MS Coupled with Multivariate Statistical Analysis to Characterize Nucleosides, Nucleobases and Amino Acids in Angelicae Sinensis Radix Obtained with Different Drying Method

Shaoqing Zhu, Sheng Guo*, Jin-ao Duan*, Dawei Qian, Hui Yan, Xiuxiu Sha, Zhenhua Zhu

Jiangsu Collaborative Innovation Center of Chinese Medicinal Resources Industrialization, and National and Local Collaborative Engineering Center of Chinese Medicinal Resources Industrialization and Formulae Innovative Medicine, Nanjing University of Chinese Medicine, Nanjing 210023, PR China, zhushaoqing1505@163.com (S. Z.); qiandw@njucm.edu.cn (D. Q.); glory-yan@163.com (H. Y.); 1023978814@qq.com (X. S.); 04040416@163.com (Z. Z.)

*Correspondence: guosheng@njucm.edu.cn (S. G.); dja@njucm.edu.cn (J. D.); Tel/Fax: +86 25 85811917 (S. G.); +86 25 85811291 (J. D.)

Table S1. Contents of the nucleosides and nucleobases in Angelica Sinensis Radix (n=3)

Sample No.[a]	Contents (mg/g, mean ± SD)																		
	**1**[b]	**2**	**3**	**4**	**5**	**6**	**7**	**8**	**9**	**10**	**11**	**12**	**13**	**14**	**15**	**16**	**17**	**18**	**Total**
1	ND[c]	ND	0.05 ± 0.00	0.04 ± 0.00	0.94 ± 0.06	0.05 ± 0.00	0.66 ± 0.01	ND	0.11 ± 0.01	0.04 ± 0.00	0.64 ± 0.04	ND	1.30 ± 0.09	0.78 ± 0.06	0.21 ± 0.02	0.05 ± 0.00	0.06 ± 0.00	0.56 ± 0.06	5.49 ± 0.16
2	0.02 ± 0.01	Trace[d]	0.09 ± 0.00	0.09 ± 0.00	0.75 ± 0.07	0.14 ± 0.01	0.48 ± 0.01	Trace	0.17 ± 0.01	0.09 ± 0.00	1.15 ± 0.05	ND	0.27 ± 0.02	0.55 ± 0.02	0.24 ± 0.01	0.11 ± 0.02	0.08 ± 0.00	0.62 ± 0.03	4.85 ± 0.15
3	0.03 ± 0.00	0.04 ± 0.01	0.16 ± 0.00	0.15 ± 0.01	0.68 ± 0.09	0.07 ± 0.01	0.67 ± 0.02	ND	0.06 ± 0.01	0.11 ± 0.01	1.31 ± 0.11	ND	1.11 ± 0.17	0.90 ± 0.06	0.19 ± 0.02	0.07 ± 0.01	0.07 ± 0.01	0.60 ± 0.07	6.21 ± 0.19
4	0.04 ± 0.00	0.05 ± 0.01	0.13 ± 0.00	0.11 ± 0.00	0.83 ± 0.04	0.14 ± 0.01	0.58 ± 0.01	0.01 ± 0.00	0.27 ± 0.01	0.09 ± 0.01	0.85 ± 0.04	ND	1.36 ± 0.12	0.69 ± 0.09	0.19 ± 0.03	0.06 ± 0.01	0.03 ± 0.01	0.48 ± 0.03	5.91 ± 0.18
5	0.05 ± 0.01	Trace	0.13 ± 0.00	0.12 ± 0.01	0.51 ± 0.05	0.34 ± 0.02	0.33 ± 0.01	0.01 ± 0.00	0.22 ± 0.01	0.15 ± 0.01	1.45 ± 0.13	ND	0.27 ± 0.03	0.35 ± 0.05	0.18 ± 0.01	0.05 ± 0.01	0.06 ± 0.01	0.43 ± 0.06	4.65 ± 0.14
6	Trace	ND	0.07 ± 0.00	0.06 ± 0.00	0.77 ± 0.07	0.11 ± 0.00	0.62 ± 0.01	ND	0.15 ± 0.00	0.07 ± 0.00	0.70 ± 0.02	ND	1.72 ± 0.23	1.04 ± 0.07	0.16 ± 0.02	0.05 ± 0.01	0.02 ± 0.00	0.48 ± 0.05	5.99 ± 0.18
7	0.04 ± 0.00	0.06 ± 0.01	0.14 ± 0.00	0.12 ± 0.01	0.72 ± 0.06	0.10 ± 0.00	0.72 ± 0.01	0.01 ± 0.00	0.17 ± 0.01	0.09 ± 0.00	0.78 ± 0.07	ND	0.61 ± 0.06	0.75 ± 0.09	0.12 ± 0.01	0.12 ± 0.02	0.04 ± 0.01	0.58 ± 0.06	5.16 ± 0.15
8	0.05 ± 0.01	0.05 ± 0.01	0.14 ± 0.00	0.13 ± 0.01	0.50 ± 0.06	0.14 ± 0.01	0.51 ± 0.00	0.01 ± 0.00	0.20 ± 0.01	0.10 ± 0.00	0.73 ± 0.05	ND	0.14 ± 0.01	0.67 ± 0.08	0.10 ± 0.01	0.08 ± 0.01	0.03 ± 0.00	0.49 ± 0.03	4.07 ± 0.12
9	ND	Trace	0.05 ± 0.00	0.04 ± 0.00	0.67 ± 0.06	0.05 ± 0.00	0.64 ± 0.01	ND	0.12 ± 0.01	0.04 ± 0.00	0.35 ± 0.03	ND	0.78 ± 0.12	0.56 ± 0.07	0.08 ± 0.01	0.05 ± 0.01	0.02 ± 0.00	0.43 ± 0.04	3.89 ± 0.12
10	0.02 ± 0.00	0.04 ± 0.02	0.12 ± 0.00	0.11 ± 0.01	0.81 ± 0.03	0.05 ± 0.00	0.76 ± 0.02	ND	0.10 ± 0.01	0.05 ± 0.00	0.65 ± 0.06	ND	1.30 ± 0.15	0.89 ± 0.06	0.16 ± 0.02	0.07 ± 0.01	0.04 ± 0.00	0.59 ± 0.01	5.76 ± 0.17
11	Trace	Trace	0.10 ± 0.00	0.09 ± 0.00	0.91 ± 0.07	0.03 ± 0.00	0.79 ± 0.01	ND	0.06 ± 0.01	0.04 ± 0.00	0.57 ± 0.06	ND	0.68 ± 0.07	0.89 ± 0.09	0.12 ± 0.01	0.11 ± 0.01	0.03 ± 0.00	0.55 ± 0.02	4.97 ± 0.15
12	0.04 ± 0.01	Trace	0.09 ± 0.00	0.08 ± 0.00	0.61 ± 0.06	0.08 ± 0.00	0.62 ± 0.01	0.01 ± 0.00	0.20 ± 0.02	0.08 ± 0.00	0.76 ± 0.02	ND	1.12 ± 0.10	0.71 ± 0.07	0.17 ± 0.01	0.17 ± 0.02	0.03 ± 0.00	0.81 ± 0.07	5.57 ± 0.17
13	0.02 ± 0.00	0.03 ± 0.00	0.09 ± 0.01	0.09 ± 0.01	0.45 ± 0.03	0.16 ± 0.01	0.45 ± 0.02	0.01 ± 0.00	0.26 ± 0.02	0.08 ± 0.00	0.55 ± 0.06	ND	0.46 ± 0.06	0.50 ± 0.06	0.26 ± 0.02	0.08 ± 0.01	0.02 ± 0.00	0.62 ± 0.02	4.15 ± 0.12
14	0.02 ± 0.00	Trace	0.14 ± 0.00	0.13 ± 0.00	0.08 ± 0.01	0.27 ± 0.01	0.21 ± 0.00	Trace	0.03 ± 0.00	0.26 ± 0.01	1.87 ± 0.04	ND	Trace	0.10 ± 0.01	0.34 ± 0.02	0.11 ± 0.01	0.04 ± 0.00	0.67 ± 0.01	4.28 ± 0.13
15	0.02 ± 0.00	ND	0.10 ± 0.00	0.09 ± 0.01	0.70 ± 0.06	0.09 ± 0.00	0.68 ± 0.01	0.00 ± 0.00	0.11 ± 0.01	0.15 ± 0.01	0.83 ± 0.06	ND	0.55 ± 0.06	0.67 ± 0.05	0.31 ± 0.02	0.07 ± 0.01	0.02 ± 0.00	0.53 ± 0.03	4.93 ± 0.15
16	0.02 ± 0.00	0.03 ± 0.01	0.10 ± 0.00	0.10 ± 0.01	0.96 ± 0.02	0.04 ± 0.00	0.87 ± 0.03	Trace	0.05 ± 0.01	0.05 ± 0.00	0.46 ± 0.03	ND	1.05 ± 0.08	0.96 ± 0.10	0.23 ± 0.01	0.09 ± 0.01	0.03 ± 0.00	0.49 ± 0.05	5.54 ± 0.17

17	Trace	Trace	0.06 ± 0.00	0.06 ± 0.01	0.82 ± 0.10	0.05 ± 0.00	0.83 ± 0.02	Trace	0.05 ± 0.01	0.03 ± 0.00	0.33 ± 0.02	ND	1.05 ± 0.08	0.82 ± 0.09	0.19 ± 0.02	0.09 ± 0.01	0.03 ± 0.00	0.41 ± 0.05	4.81 ± 0.14
18	0.02 ± 0.00	0.03 ± 0.01	0.10 ± 0.00	0.10 ± 0.00	1.05 ± 0.12	0.04 ± 0.00	0.91 ± 0.01	Trace	0.08 ± 0.01	0.04 ± 0.00	0.37 ± 0.05	ND	1.35 ± 0.12	0.94 ± 0.10	0.29 ± 0.03	0.13 ± 0.02	0.03 ± 0.00	0.51 ± 0.06	6.00 ± 0.18
19	ND	ND	0.01 ± 0.00	0.01 ± 0.00	0.76 ± 0.04	0.01 ± 0.00	0.61 ± 0.01	ND	0.01 ± 0.00	0.01 ± 0.00	0.13 ± 0.01	ND	0.70 ± 0.11	0.87 ± 0.12	0.06 ± 0.01	0.05 ± 0.01	0.03 ± 0.01	0.20 ± 0.02	3.46 ± 0.10
20	0.02 ± 0.00	ND	0.08 ± 0.00	0.07 ± 0.00	0.88 ± 0.09	0.06 ± 0.00	0.53 ± 0.00	ND	0.07 ± 0.01	0.02 ± 0.00	0.37 ± 0.03	ND	1.25 ± 0.12	0.71 ± 0.13	0.10 ± 0.01	0.10 ± 0.01	Trace	0.19 ± 0.02	4.46 ± 0.13
21	Trace	ND	0.04 ± 0.00	0.04 ± 0.00	0.81 ± 0.04	0.04 ± 0.00	0.72 ± 0.01	ND	0.03 ± 0.00	0.02 ± 0.00	0.19 ± 0.00	ND	1.94 ± 0.22	0.94 ± 0.13	0.12 ± 0.01	0.10 ± 0.01	0.03 ± 0.01	0.31 ± 0.03	5.32 ± 0.16
22	ND	ND	0.03 ± 0.00	0.03 ± 0.00	0.84 ± 0.12	0.04 ± 0.00	0.43 ± 0.00	ND	0.05 ± 0.00	0.03 ± 0.00	0.31 ± 0.03	ND	0.65 ± 0.10	0.76 ± 0.12	0.08 ± 0.01	0.09 ± 0.01	0.03 ± 0.01	0.21 ± 0.02	3.60 ± 0.11
23	ND	ND	0.02 ± 0.00	0.02 ± 0.00	0.79 ± 0.10	0.03 ± 0.00	0.43 ± 0.01	ND	0.03 ± 0.00	0.01 ± 0.00	0.21 ± 0.00	ND	0.78 ± 0.04	0.83 ± 0.09	0.06 ± 0.00	0.06 ± 0.01	0.02 ± 0.00	0.20 ± 0.03	3.49 ± 0.10
24	ND	Trace	0.03 ± 0.00	0.03 ± 0.00	0.10 ± 0.04	0.26 ± 0.01	0.09 ± 0.00	0.01 ± 0.00	0.05 ± 0.01	0.20 ± 0.01	0.44 ± 0.05	ND	0.21 ± 0.02	0.05 ± 0.01	0.30 ± 0.03	0.22 ± 0.03	0.10 ± 0.01	0.62 ± 0.06	2.70 ± 0.08
25	Trace	Trace	0.02 ± 0.00	0.02 ± 0.00	0.47 ± 0.05	0.05 ± 0.00	0.49 ± 0.01	ND	0.10 ± 0.01	0.02 ± 0.00	0.17 ± 0.01	ND	0.47 ± 0.06	0.81 ± 0.11	0.18 ± 0.01	0.16 ± 0.02	0.05 ± 0.01	0.47 ± 0.06	3.48 ± 0.10
26	ND	ND	0.03 ± 0.00	0.03 ± 0.00	0.66 ± 0.04	0.03 ± 0.00	0.62 ± 0.01	ND	0.05 ± 0.01	0.02 ± 0.00	0.17 ± 0.02	ND	0.92 ± 0.11	1.11 ± 0.12	0.17 ± 0.02	0.11 ± 0.01	0.03 ± 0.00	0.37 ± 0.04	4.32 ± 0.13
27	ND	ND	0.04 ± 0.00	0.04 ± 0.00	0.60 ± 0.08	0.03 ± 0.00	0.58 ± 0.01	ND	0.04 ± 0.01	0.01 ± 0.00	0.12 ± 0.02	ND	0.64 ± 0.07	1.15 ± 0.17	0.22 ± 0.02	0.13 ± 0.02	0.03 ± 0.00	0.48 ± 0.05	4.12 ± 0.12
28	Trace	ND	0.02 ± 0.00	0.02 ± 0.00	0.40 ± 0.04	0.04 ± 0.00	0.36 ± 0.01	ND	0.02 ± 0.00	0.04 ± 0.00	0.20 ± 0.02	ND	0.49 ± 0.08	0.46 ± 0.06	0.15 ± 0.00	0.11 ± 0.01	0.02 ± 0.00	0.30 ± 0.03	2.61 ± 0.08
29	ND	ND	0.02 ± 0.00	0.03 ± 0.00	0.55 ± 0.07	0.14 ± 0.00	0.41 ± 0.00	ND	0.02 ± 0.00	0.13 ± 0.00	1.09 ± 0.04	ND	0.13 ± 0.02	0.50 ± 0.07	0.22 ± 0.03	0.12 ± 0.01	0.04 ± 0.01	0.24 ± 0.02	3.64 ± 0.11
30	0.02 ± 0.00	ND	0.02 ± 0.00	0.02 ± 0.00	0.57 ± 0.08	0.05 ± 0.01	0.42 ± 0.01	Trace	0.10 ± 0.01	0.08 ± 0.01	0.48 ± 0.02	ND	0.24 ± 0.03	0.48 ± 0.07	0.23 ± 0.03	0.12 ± 0.01	0.03 ± 0.00	0.28 ± 0.02	3.15 ± 0.09
31	Trace	ND	0.01 ± 0.00	0.01 ± 0.00	0.07 ± 0.01	0.04 ± 0.01	0.10 ± 0.01	ND	Trace	0.07 ± 0.00	0.22 ± 0.02	ND	ND	0.43 ± 0.05	0.11 ± 0.01	0.08 ± 0.01	Trace	0.15 ± 0.02	1.30 ± 0.04
32	Trace	ND	0.02 ± 0.00	0.03 ± 0.00	0.35 ± 0.04	0.03 ± 0.00	0.21 ± 0.01	ND	0.02 ± 0.00	0.04 ± 0.00	0.03 ± 0.00	ND	Trace	0.95 ± 0.08	0.19 ± 0.02	0.12 ± 0.01	0.03 ± 0.00	0.25 ± 0.03	2.26 ± 0.07
33	ND	ND	0.07 ± 0.00	0.07 ± 0.01	1.04 ± 0.10	0.09 ± 0.01	0.37 ± 0.00	Trace	0.03 ± 0.00	0.01 ± 0.00	0.62 ± 0.09	ND	0.26 ± 0.03	0.43 ± 0.05	0.07 ± 0.01	0.11 ± 0.01	0.06 ± 0.01	0.24 ± 0.01	3.47 ± 0.10
34	Trace	ND	0.06 ± 0.01	0.06 ± 0.00	0.87 ± 0.09	0.05 ± 0.01	0.53 ± 0.01	ND	0.02 ± 0.00	0.02 ± 0.00	0.18 ± 0.01	ND	ND	0.59 ± 0.05	0.09 ± 0.01	0.12 ± 0.01	0.07 ± 0.01	0.22 ± 0.02	2.89 ± 0.09

35	Trace	ND	0.07 ± 0.00	0.05 ± 0.00	1.16 ± 0.16	0.06 ± 0.00	0.48 ± 0.00	ND	0.01 ± 0.00	0.01 ± 0.00	0.20 ± 0.03	ND	0.12 ± 0.02	0.70 ± 0.08	0.07 ± 0.01	0.09 ± 0.01	0.07 ± 0.01	0.17 ± 0.02	3.26 ± 0.10
36	ND	ND	0.04 ± 0.00	0.06 ± 0.00	0.53 ± 0.05	0.01 ± 0.00	0.37 ± 0.05	ND	Trace	0.00 ± 0.00	0.03 ± 0.00	ND	0.11 ± 0.02	0.51 ± 0.04	0.06 ± 0.01	0.37 ± 0.04	0.04 ± 0.01	0.11 ± 0.01	2.26 ± 0.07
37	Trace	Trace	0.03 ± 0.00	0.03 ± 0.00	0.64 ± 0.06	0.02 ± 0.00	0.51 ± 0.02	ND	0.04 ± 0.01	0.05 ± 0.00	0.11 ± 0.01	ND	0.08 ± 0.01	0.41 ± 0.05	0.04 ± 0.01	0.35 ± 0.05	0.02 ± 0.00	0.08 ± 0.01	2.41 ± 0.07
38	0.01 ± 0.00	Trace	0.07 ± 0.00	0.06 ± 0.01	0.61 ± 0.09	0.11 ± 0.01	0.50 ± 0.00	ND	0.05 ± 0.01	0.06 ± 0.00	0.31 ± 0.04	ND	0.35 ± 0.04	0.51 ± 0.07	0.08 ± 0.01	0.78 ± 0.09	0.07 ± 0.01	0.17 ± 0.01	3.75 ± 0.11
39	ND	ND	Trace	ND	ND	0.08 ± 0.01	0.00 ± 0.00	ND	ND	0.09 ± 0.00	0.15 ± 0.01	ND	ND	ND	0.04 ± 0.00	1.16 ± 0.18	0.07 ± 0.01	0.22 ± 0.03	1.80 ± 0.05
40	0.02 ± 0.00	Trace	0.05 ± 0.00	0.05 ± 0.01	0.87 ± 0.09	0.22 ± 0.01	0.56 ± 0.01	ND	0.01 ± 0.00	0.03 ± 0.00	0.33 ± 0.03	ND	0.30 ± 0.04	0.50 ± 0.01	0.06 ± 0.00	0.53 ± 0.06	0.05 ± 0.01	0.12 ± 0.02	3.70 ± 0.11
41	0.03 ± 0.00	Trace	0.08 ± 0.00	0.08 ± 0.00	0.90 ± 0.10	0.12 ± 0.00	0.64 ± 0.02	ND	Trace	0.10 ± 0.00	0.88 ± 0.03	ND	0.38 ± 0.04	0.58 ± 0.08	0.09 ± 0.01	0.76 ± 0.04	0.07 ± 0.01	0.17 ± 0.02	4.87 ± 0.15
42	ND	ND	0.01 ± 0.00	0.01 ± 0.00	0.37 ± 0.05	0.02 ± 0.00	0.50 ± 0.05	ND	Trace	ND	0.09 ± 0.01	ND	ND	0.14 ± 0.02	0.08 ± 0.01	0.72 ± 0.07	0.14 ± 0.02	0.24 ± 0.03	2.31 ± 0.07
43	0.02 ± 0.00	0.02 ± 0.00	0.12 ± 0.00	0.09 ± 0.00	1.24 ± 0.19	0.04 ± 0.00	0.56 ± 0.02	ND	0.02 ± 0.00	0.01 ± 0.00	0.09 ± 0.01	ND	0.52 ± 0.04	0.60 ± 0.07	0.07 ± 0.00	0.65 ± 0.02	0.06 ± 0.01	0.15 ± 0.00	4.24 ± 0.13
44	Trace	Trace	0.11 ± 0.00	0.08 ± 0.00	1.02 ± 0.06	0.01 ± 0.00	1.02 ± 0.01	ND	0.01 ± 0.00	Trace	0.04 ± 0.01	ND	0.14 ± 0.01	0.91 ± 0.05	0.06 ± 0.01	0.38 ± 0.05	0.08 ± 0.01	0.33 ± 0.04	4.17 ± 0.13
45	0.02 ± 0.00	0.02 ± 0.00	0.09 ± 0.00	0.08 ± 0.01	1.06 ± 0.04	0.07 ± 0.00	0.97 ± 0.03	ND	0.02 ± 0.00	0.02 ± 0.00	0.29 ± 0.03	ND	1.51 ± 0.17	1.42 ± 0.13	0.08 ± 0.01	0.20 ± 0.03	0.07 ± 0.01	0.24 ± 0.02	6.15 ± 0.18
46	ND	ND	0.00 ± 0.00	0.14 ± 0.01	0.11 ± 0.03	0.01 ± 0.00	0.44 ± 0.01	ND	ND	ND	0.04 ± 0.00	ND	ND	0.03 ± 0.00	0.07 ± 0.00	0.24 ± 0.03	0.54 ± 0.06	0.21 ± 0.01	1.82 ± 0.05
47	Trace	ND	0.03 ± 0.00	0.03 ± 0.00	0.86 ± 0.03	0.03 ± 0.00	0.86 ± 0.01	ND	0.02 ± 0.00	0.01 ± 0.00	0.18 ± 0.02	ND	0.42 ± 0.04	0.81 ± 0.11	0.08 ± 0.01	0.27 ± 0.03	0.08 ± 0.01	0.24 ± 0.02	3.92 ± 0.12
48	Trace	Trace	0.05 ± 0.00	0.05 ± 0.00	0.37 ± 0.05	0.13 ± 0.00	0.54 ± 0.01	Trace	0.11 ± 0.01	0.12 ± 0.00	0.69 ± 0.02	ND	0.26 ± 0.03	0.46 ± 0.06	0.06 ± 0.01	0.16 ± 0.02	0.06 ± 0.01	0.19 ± 0.02	3.26 ± 0.10
49	0.02 ± 0.00	Trace	0.05 ± 0.00	0.04 ± 0.01	0.30 ± 0.02	0.44 ± 0.01	0.14 ± 0.01	Trace	0.05 ± 0.00	0.11 ± 0.00	0.37 ± 0.01	ND	ND	0.02 ± 0.00	0.05 ± 0.01	0.12 ± 0.01	0.03 ± 0.00	0.13 ± 0.02	1.86 ± 0.06
50	ND	ND	ND	ND	ND	0.22 ± 0.00	0.00 ± 0.00	ND	ND	Trace	0.13 ± 0.01	ND	ND	ND	0.06 ± 0.00	0.14 ± 0.01	0.05 ± 0.01	0.19 ± 0.02	0.79 ± 0.02
Average	0.01 ± 0.01	0.01 ± 0.02	0.06 ± 0.04	0.06 ± 0.04	0.65 ± 0.31	0.09 ± 0.09	0.53 ± 0.23	-	0.07 ± 0.07	0.06 ± 0.06	0.46 ± 0.40	-	0.56 ± 0.52	0.63 ± 0.32	0.14 ± 0.08	0.21 ± 0.23	0.06 ± 0.07	0.35 ± 0.18	

[a] The sample No. is same as Table 3; [b] The analytes is same as in Table 1; [c] Not detected; [d] Under the LOQ.

Table S2. Contents of the amino acids in Angelica Sinensis Radix (n=3)

Sample No.[a]	Contents (mg/g, mean ± SD)																					
	**19**[b]	**20**	**21**	**22**	**23**	**24**	**25**	**26**	**27**	**28**	**29**	**30**	**31**	**32**	**33**	**34**	**35**	**36**	**37**	**38**	**39**	**40**	**Total**
1	0.19 ± 0.01	0.17 ± 0.00	0.26 ± 0.01	0.71 ± 0.02	2.09 ± 0.05	0.05 ± 0.00	0.79 ± 0.05	0.30 ± 0.01	ND[c]	0.22 ± 0.01	1.78 ± 0.04	0.07 ± 0.01	0.21 ± 0.01	1.47 ± 0.13	3.16 ± 0.08	3.44 ± 0.16	0.30 ± 0.02	0.37 ± 0.02	0.85 ± 0.03	78.68 ± 3.45	3.86 ± 0.06	1.22 ± 0.17	100.17 ± 3.01
2	0.28 ± 0.02	0.23 ± 0.01	0.44 ± 0.01	0.66 ± 0.02	3.32 ± 0.13	0.08 ± 0.01	1.71 ± 0.08	0.37 ± 0.01	ND	0.35 ± 0.02	1.92 ± 0.03	0.08 ± 0.01	0.40 ± 0.04	2.21 ± 0.20	2.32 ± 0.16	2.27 ± 0.14	0.37 ± 0.03	0.75 ± 0.06	1.36 ± 0.02	85.30 ± 2.40	4.16 ± 0.27	2.56 ± 0.17	111.14 ± 3.33
3	0.31 ± 0.01	0.24 ± 0.01	0.33 ± 0.01	0.59 ± 0.01	2.40 ± 0.06	0.08 ± 0.01	1.11 ± 0.02	0.33 ± 0.01	ND	0.40 ± 0.04	1.69 ± 0.02	0.06 ± 0.00	0.50 ± 0.05	2.12 ± 0.07	2.01 ± 0.14	2.21 ± 0.20	0.46 ± 0.05	1.15 ± 0.07	1.58 ± 0.05	81.35 ± 4.27	3.76 ± 0.08	0.94 ± 0.07	103.62 ± 3.11
4	0.29 ± 0.02	0.26 ± 0.01	0.37 ± 0.03	0.63 ± 0.01	2.72 ± 0.17	0.11 ± 0.00	4.15 ± 0.07	0.53 ± 0.02	ND	0.40 ± 0.02	5.20 ± 0.25	0.09 ± 0.00	0.54 ± 0.04	2.54 ± 0.08	4.11 ± 0.44	4.54 ± 0.15	0.41 ± 0.04	1.08 ± 0.04	3.55 ± 0.34	61.30 ± 0.30	3.44 ± 0.10	5.34 ± 0.55	101.60 ± 3.05
5	0.37 ± 0.02	0.27 ± 0.01	0.47 ± 0.01	0.68 ± 0.02	3.61 ± 0.12	0.10 ± 0.00	4.55 ± 0.12	0.56 ± 0.06	ND	0.38 ± 0.04	6.63 ± 0.30	Trace[d]	0.60 ± 0.06	3.30 ± 0.26	2.86 ± 0.25	3.10 ± 0.17	0.52 ± 0.07	0.44 ± 0.07	3.87 ± 0.46	59.23 ± 2.93	3.41 ± 0.15	6.17 ± 0.67	101.11 ± 3.03
6	0.24 ± 0.00	0.20 ± 0.01	0.28 ± 0.01	0.46 ± 0.02	2.85 ± 0.04	0.05 ± 0.01	3.72 ± 0.19	0.44 ± 0.00	ND	0.24 ± 0.03	3.79 ± 0.17	Trace	0.42 ± 0.01	3.44 ± 0.27	4.14 ± 0.24	4.57 ± 0.17	0.63 ± 0.08	0.92 ± 0.06	2.36 ± 0.13	53.52 ± 1.73	1.94 ± 0.13	4.14 ± 0.36	88.34 ± 2.65
7	0.43 ± 0.03	0.26 ± 0.01	0.41 ± 0.01	0.58 ± 0.02	2.40 ± 0.03	0.04 ± 0.00	3.39 ± 0.22	0.58 ± 0.04	ND	0.47 ± 0.01	4.24 ± 0.33	ND	0.53 ± 0.06	1.34 ± 0.12	1.14 ± 0.07	1.22 ± 0.11	0.68 ± 0.08	ND	1.97 ± 0.20	45.37 ± 1.06	3.08 ± 0.14	2.21 ± 0.25	70.33 ± 2.11
8	0.52 ± 0.02	0.32 ± 0.01	0.52 ± 0.02	0.64 ± 0.03	3.05 ± 0.13	0.11 ± 0.01	2.67 ± 0.07	0.54 ± 0.00	ND	0.58 ± 0.08	4.17 ± 0.34	Trace	0.46 ± 0.07	2.02 ± 0.11	3.03 ± 0.21	3.03 ± 0.11	0.73 ± 0.11	0.72 ± 0.07	1.48 ± 0.13	42.38 ± 1.53	2.90 ± 0.10	3.17 ± 0.36	73.07 ± 2.19
9	0.23 ± 0.00	0.15 ± 0.00	0.24 ± 0.00	0.34 ± 0.02	1.99 ± 0.12	Trace	2.79 ± 0.03	0.40 ± 0.03	ND	0.18 ± 0.02	3.77 ± 0.49	ND	0.32 ± 0.05	1.52 ± 0.19	2.59 ± 0.19	2.63 ± 0.12	0.82 ± 0.06	0.71 ± 0.12	2.26 ± 0.22	32.55 ± 2.44	1.42 ± 0.09	2.08 ± 0.18	56.98 ± 1.71
10	0.22 ± 0.01	0.17 ± 0.00	0.22 ± 0.00	0.55 ± 0.01	2.23 ± 0.07	0.07 ± 0.01	1.71 ± 0.11	0.33 ± 0.02	ND	0.32 ± 0.03	2.10 ± 0.16	Trace	0.32 ± 0.07	1.94 ± 0.18	2.57 ± 0.17	2.56 ± 0.15	0.50 ± 0.10	0.94 ± 0.09	0.60 ± 0.03	53.75 ± 1.59	3.10 ± 0.02	1.25 ± 0.14	75.46 ± 2.26
11	0.30 ± 0.01	0.20 ± 0.00	0.29 ± 0.01	0.47 ± 0.01	2.00 ± 0.07	0.05 ± 0.00	0.72 ± 0.05	0.31 ± 0.02	ND	0.29 ± 0.03	1.32 ± 0.12	Trace	0.40 ± 0.06	3.73 ± 0.35	1.58 ± 0.03	1.58 ± 0.08	0.58 ± 0.08	0.85 ± 0.09	0.57 ± 0.04	51.80 ± 1.54	2.49 ± 0.05	0.63 ± 0.06	70.15 ± 2.10
12	0.21 ± 0.02	0.23 ± 0.01	0.37 ± 0.01	0.83 ± 0.03	3.06 ± 0.04	0.08 ± 0.01	1.23 ± 0.05	0.35 ± 0.03	ND	0.28 ± 0.04	1.42 ± 0.14	Trace	0.32 ± 0.03	0.37 ± 0.04	4.11 ± 0.06	4.10 ± 0.15	0.72 ± 0.12	1.58 ± 0.16	0.90 ± 0.16	73.08 ± 2.37	5.20 ± 0.29	1.25 ± 0.13	99.68 ± 2.99
13	0.45 ± 0.02	0.30 ± 0.01	0.54 ± 0.04	0.84 ± 0.03	3.17 ± 0.09	0.10 ± 0.01	3.50 ± 0.14	0.64 ± 0.07	ND	0.53 ± 0.06	5.00 ± 0.10	Trace	0.41 ± 0.06	2.12 ± 0.12	3.75 ± 0.10	3.86 ± 0.07	0.66 ± 0.06	0.82 ± 0.08	1.92 ± 0.23	48.31 ± 2.76	4.13 ± 0.20	4.94 ± 0.08	85.97 ± 2.58
14	0.44 ± 0.03	0.33 ± 0.00	0.56 ± 0.02	0.87 ± 0.01	3.52 ± 0.09	0.10 ± 0.01	2.11 ± 0.06	0.45 ± 0.01	ND	0.62 ± 0.05	3.92 ± 0.37	Trace	0.52 ± 0.06	2.52 ± 0.19	3.44 ± 0.12	3.61 ± 0.14	0.47 ± 0.06	0.45 ± 0.06	1.42 ± 0.17	58.20 ± 0.38	5.03 ± 0.13	2.61 ± 0.25	91.21 ± 2.74
15	0.33 ± 0.01	0.25 ± 0.01	0.35 ± 0.00	0.62 ± 0.02	2.06 ± 0.08	0.07 ± 0.01	4.78 ± 0.16	0.67 ± 0.07	ND	0.28 ± 0.03	6.75 ± 0.54	ND	0.40 ± 0.04	2.60 ± 0.05	2.88 ± 0.12	2.85 ± 0.13	0.49 ± 0.08	0.68 ± 0.10	2.80 ± 0.29	44.24 ± 0.49	3.18 ± 0.08	6.68 ± 0.35	82.97 ± 2.49
16	0.39 ± 0.03	0.22 ± 0.01	0.32 ± 0.00	0.43 ± 0.01	2.59 ± 0.12	0.04 ± 0.01	2.25 ± 0.03	0.47 ± 0.03	ND	0.39 ± 0.03	3.32 ± 0.06	0.04 ± 0.00	0.54 ± 0.03	0.57 ± 0.02	2.11 ± 0.12	2.24 ± 0.13	0.64 ± 0.10	0.74 ± 0.11	2.12 ± 0.17	42.79 ± 1.47	2.29 ± 0.11	2.45 ± 0.19	66.97 ± 2.01

17	0.34 ± 0.00	0.22 ± 0.01	0.40 ± 0.00	0.45 ± 0.01	2.99 ± 0.14	0.02 ± 0.00	3.26 ± 0.06	0.57 ± 0.02	ND	0.36 ± 0.03	4.23 ± 0.40	0.05 ± 0.01	0.63 ± 0.07	0.94 ± 0.06	3.77 ± 0.10	3.98 ± 0.05	0.34 ± 0.04	0.68 ± 0.08	1.95 ± 0.24	35.25 ± 2.72	2.18 ± 0.15	2.60 ± 1.15	65.22 ± 1.96
18	0.50 ± 0.01	0.28 ± 0.01	0.44 ± 0.02	0.48 ± 0.01	3.17 ± 0.18	0.07 ± 0.01	2.48 ± 0.17	0.55 ± 0.03	ND	0.42 ± 0.06	3.15 ± 0.13	Trace	0.56 ± 0.06	0.75 ± 0.08	2.10 ± 0.18	2.08 ± 0.13	0.35 ± 0.05	0.87 ± 0.12	1.00 ± 0.11	46.80 ± 0.67	2.68 ± 0.11	2.37 ± 0.31	71.09 ± 2.13
19	0.08 ± 0.00	0.06 ± 0.00	0.12 ± 0.00	0.22 ± 0.00	2.28 ± 0.07	0.03 ± 0.00	0.54 ± 0.05	0.13 ± 0.01	ND	0.11 ± 0.02	1.13 ± 0.12	ND	0.18 ± 0.02	0.50 ± 0.06	0.97 ± 0.04	0.94 ± 0.04	Trace	0.41 ± 0.06	0.37 ± 0.05	32.59 ± 0.70	1.09 ± 0.07	0.27 ± 0.04	42.01 ± 1.26
20	0.12 ± 0.00	0.09 ± 0.00	0.16 ± 0.00	0.24 ± 0.00	2.54 ± 0.11	0.05 ± 0.01	0.90 ± 0.03	0.19 ± 0.00	ND	0.12 ± 0.02	1.05 ± 0.05	ND	0.21 ± 0.03	0.58 ± 0.08	1.48 ± 0.03	1.62 ± 0.07	0.41 ± 0.11	0.27 ± 0.04	0.23 ± 0.03	31.91 ± 1.76	1.27 ± 0.04	0.28 ± 0.03	43.72 ± 1.31
21	0.16 ± 0.01	0.12 ± 0.00	0.17 ± 0.01	0.54 ± 0.02	2.51 ± 0.03	0.04 ± 0.00	0.42 ± 0.03	0.16 ± 0.00	ND	0.29 ± 0.01	0.81 ± 0.05	Trace	0.46 ± 0.05	1.18 ± 0.14	2.93 ± 0.10	3.10 ± 0.13	0.46 ± 0.05	0.86 ± 0.05	0.48 ± 0.05	52.09 ± 2.09	2.30 ± 0.23	0.29 ± 0.03	69.36 ± 2.08
22	0.12 ± 0.00	0.08 ± 0.01	0.12 ± 0.00	0.29 ± 0.00	2.42 ± 0.01	0.03 ± 0.01	2.91 ± 0.14	0.18 ± 0.02	ND	0.09 ± 0.01	1.03 ± 0.04	Trace	0.20 ± 0.02	0.22 ± 0.03	0.56 ± 0.06	0.66 ± 0.03	0.33 ± 0.04	0.34 ± 0.04	0.45 ± 0.05	36.29 ± 1.10	1.31 ± 0.04	0.33 ± 0.06	47.99 ± 1.44
23	0.11 ± 0.01	0.08 ± 0.01	0.11 ± 0.00	0.30 ± 0.00	1.90 ± 0.07	0.04 ± 0.00	0.67 ± 0.07	0.14 ± 0.02	ND	0.17 ± 0.02	1.06 ± 0.11	Trace	0.15 ± 0.02	4.22 ± 0.47	0.89 ± 0.06	0.96 ± 0.11	0.45 ± 0.07	0.15 ± 0.02	0.20 ± 0.03	29.92 ± 0.82	1.17 ± 0.13	0.35 ± 0.05	43.03 ± 1.29
24	0.34 ± 0.00	0.26 ± 0.02	0.33 ± 0.01	0.85 ± 0.04	3.26 ± 0.03	0.13 ± 0.01	0.77 ± 0.03	0.30 ± 0.04	ND	0.28 ± 0.03	1.01 ± 0.15	Trace	0.30 ± 0.04	0.84 ± 0.06	1.80 ± 0.09	1.89 ± 0.05	0.41 ± 0.05	0.26 ± 0.03	4.18 ± 0.55	58.50 ± 1.49	3.16 ± 0.25	0.66 ± 0.07	79.53 ± 2.39
25	0.14 ± 0.00	0.11 ± 0.00	0.17 ± 0.01	0.58 ± 0.01	2.77 ± 0.04	0.05 ± 0.01	0.35 ± 0.00	0.17 ± 0.02	ND	0.19 ± 0.02	0.99 ± 0.02	ND	0.12 ± 0.02	1.58 ± 0.06	2.19 ± 0.23	2.32 ± 0.12	0.63 ± 0.08	0.21 ± 0.03	0.29 ± 0.04	38.73 ± 1.13	1.68 ± 0.08	0.41 ± 0.05	53.69 ± 1.61
26	0.15 ± 0.00	0.11 ± 0.00	0.17 ± 0.00	0.39 ± 0.00	2.86 ± 0.12	0.04 ± 0.01	0.96 ± 0.03	0.19 ± 0.02	ND	0.15 ± 0.02	1.30 ± 0.14	Trace	0.12 ± 0.07	0.14 ± 0.02	0.82 ± 0.02	0.97 ± 0.06	0.47 ± 0.06	0.33 ± 0.05	0.17 ± 0.02	33.82 ± 1.70	1.15 ± 0.13	0.30 ± 0.04	44.62 ± 1.34
27	0.17 ± 0.00	0.13 ± 0.01	0.20 ± 0.00	0.41 ± 0.01	2.87 ± 0.09	0.05 ± 0.01	0.85 ± 0.05	0.21 ± 0.02	ND	0.16 ± 0.02	0.91 ± 0.10	0.04 ± 0.01	0.26 ± 0.03	1.07 ± 0.08	1.28 ± 0.04	1.27 ± 0.03	0.52 ± 0.08	1.21 ± 0.13	0.66 ± 0.08	39.30 ± 1.12	1.65 ± 0.04	0.42 ± 0.06	53.62 ± 1.61
28	0.11 ± 0.01	0.08 ± 0.00	0.12 ± 0.00	0.43 ± 0.03	2.09 ± 0.02	0.03 ± 0.00	0.33 ± 0.04	0.11 ± 0.01	ND	0.13 ± 0.02	0.88 ± 0.12	Trace	0.11 ± 0.01	0.08 ± 0.02	0.66 ± 0.03	0.66 ± 0.02	0.63 ± 0.08	0.34 ± 0.04	0.36 ± 0.04	27.29 ± 0.85	1.19 ± 0.05	0.24 ± 0.03	35.86 ± 1.08
29	0.19 ± 0.01	0.15 ± 0.01	0.25 ± 0.00	0.49 ± 0.03	1.93 ± 0.10	0.05 ± 0.01	1.87 ± 0.09	0.25 ± 0.02	ND	0.24 ± 0.02	1.07 ± 0.13	Trace	0.21 ± 0.04	6.73 ± 0.35	1.67 ± 0.09	1.88 ± 0.05	0.53 ± 0.05	0.50 ± 0.06	0.89 ± 0.10	40.86 ± 0.80	2.09 ± 0.03	0.42 ± 0.06	62.28 ± 1.87
30	0.18 ± 0.02	0.11 ± 0.01	0.05 ± 0.01	0.38 ± 0.06	2.52 ± 0.31	0.02 ± 0.00	2.42 ± 0.08	0.11 ± 0.01	ND	0.09 ± 0.01	0.56 ± 0.06	Trace	Trace	0.14 ± 0.02	0.57 ± 0.08	0.56 ± 0.06	0.37 ± 0.05	0.16 ± 0.02	0.48 ± 0.05	47.06 ± 0.24	1.98 ± 0.09	2.02 ± 0.28	59.78 ± 1.79
31	0.07 ± 0.01	0.05 ± 0.00	0.07 ± 0.00	0.13 ± 0.01	2.37 ± 0.20	0.01 ± 0.00	0.69 ± 0.04	0.10 ± 0.01	ND	0.07 ± 0.02	0.60 ± 0.04	ND	Trace	0.20 ± 0.03	0.59 ± 0.04	0.60 ± 0.08	0.19 ± 0.02	0.22 ± 0.03	0.30 ± 0.03	24.25 ± 0.83	0.80 ± 0.10	0.33 ± 0.05	31.67 ± 0.95
32	0.48 ± 0.02	0.18 ± 0.01	0.11 ± 0.01	0.24 ± 0.02	1.92 ± 0.18	0.01 ± 0.00	4.19 ± 0.35	0.43 ± 0.04	ND	0.17 ± 0.01	1.32 ± 0.12	Trace	Trace	1.04 ± 0.08	2.16 ± 0.10	1.86 ± 0.19	0.30 ± 0.04	0.83 ± 0.08	0.39 ± 0.05	40.66 ± 0.87	1.82 ± 0.16	0.93 ± 0.10	59.02 ± 1.77
33	0.14 ± 0.01	0.08 ± 0.00	0.08 ± 0.00	0.12 ± 0.01	2.48 ± 0.07	0.03 ± 0.00	1.61 ± 0.09	0.16 ± 0.01	ND	0.21 ± 0.02	1.50 ± 0.16	ND	0.29 ± 0.04	0.48 ± 0.06	0.53 ± 0.03	0.57 ± 0.01	0.20 ± 0.02	0.46 ± 0.04	0.36 ± 0.05	41.82 ± 1.10	1.22 ± 0.06	0.75 ± 0.08	53.07 ± 1.59
34	0.10 ± 0.00	0.06 ± 0.00	0.09 ± 0.00	0.29 ± 0.03	1.34 ± 0.05	0.02 ± 0.00	0.49 ± 0.01	0.11 ± 0.01	ND	0.19 ± 0.02	1.29 ± 0.13	Trace	Trace	5.53 ± 0.49	1.07 ± 0.09	1.20 ± 0.14	0.39 ± 0.06	0.37 ± 0.04	0.18 ± 0.02	42.80 ± 0.67	1.37 ± 0.05	0.56 ± 0.08	57.45 ± 1.72

35	0.12 ± 0.01	0.08 ± 0.00	0.09 ± 0.00	0.15 ± 0.00	3.08 ± 0.05	0.02 ± 0.00	2.59 ± 0.01	0.22 ± 0.01	ND	0.13 ± 0.01	1.37 ± 0.13	Trace	0.19 ± 0.03	0.74 ± 0.11	0.94 ± 0.10	1.06 ± 0.02	0.17 ± 0.03	0.15 ± 0.02	0.15 ± 0.02	32.45 ± 1.32	0.92 ± 0.06	0.38 ± 0.05	45.00 ± 1.35
36	0.05 ± 0.00	0.05 ± 0.00	0.07 ± 0.01	0.19 ± 0.01	2.07 ± 0.10	0.02 ± 0.00	1.09 ± 0.02	0.10 ± 0.01	ND	0.07 ± 0.01	0.97 ± 0.06	0.03 ± 0.00	0.11 ± 0.01	3.22 ± 0.13	0.58 ± 0.03	0.67 ± 0.05	0.57 ± 0.07	0.25 ± 0.03	0.31 ± 0.04	21.43 ± 0.09	0.82 ± 0.08	0.43 ± 0.05	33.10 ± 0.99
37	0.15 ± 0.00	0.06 ± 0.00	0.02 ± 0.00	0.16 ± 0.01	1.08 ± 0.04	0.02 ± 0.00	0.96 ± 0.03	0.05 ± 0.01	ND	0.10 ± 0.01	0.66 ± 0.07	0.03 ± 0.00	0.15 ± 0.02	0.33 ± 0.02	0.08 ± 0.01	0.11 ± 0.02	0.18 ± 0.02	Trace	0.28 ± 0.03	14.34 ± 0.48	0.47 ± 0.02	0.38 ± 0.03	19.59 ± 0.59
38	0.18 ± 0.00	0.14 ± 0.00	0.15 ± 0.00	0.42 ± 0.01	3.16 ± 0.07	0.04 ± 0.00	0.70 ± 0.02	0.18 ± 0.01	ND	0.29 ± 0.03	1.28 ± 0.12	ND	0.23 ± 0.02	0.16 ± 0.02	0.91 ± 0.01	0.91 ± 0.08	0.68 ± 0.07	0.23 ± 0.03	0.27 ± 0.04	28.83 ± 0.54	1.01 ± 0.08	0.62 ± 0.01	40.40 ± 1.21
39	0.31 ± 0.01	0.19 ± 0.01	0.17 ± 0.01	0.55 ± 0.04	2.68 ± 0.05	0.02 ± 0.00	3.89 ± 0.05	0.48 ± 0.01	ND	0.18 ± 0.02	1.71 ± 0.24	0.08 ± 0.01	0.15 ± 0.02	4.59 ± 0.40	0.05 ± 0.01	0.04 ± 0.00	0.24 ± 0.02	Trace	1.52 ± 0.14	34.15 ± 1.05	2.33 ± 0.02	6.47 ± 0.45	59.79 ± 1.79
40	0.11 ± 0.01	0.09 ± 0.01	0.09 ± 0.00	0.27 ± 0.01	2.88 ± 0.08	0.02 ± 0.00	1.19 ± 0.01	0.12 ± 0.01	ND	0.16 ± 0.01	0.97 ± 0.07	Trace	0.25 ± 0.03	1.40 ± 0.19	0.79 ± 0.08	0.86 ± 0.04	0.65 ± 0.06	0.21 ± 0.02	0.47 ± 0.02	23.21 ± 0.53	1.10 ± 0.02	0.82 ± 0.08	35.65 ± 1.07
41	0.25 ± 0.00	0.19 ± 0.01	0.24 ± 0.00	0.60 ± 0.02	2.70 ± 0.07	0.06 ± 0.01	1.60 ± 0.01	0.25 ± 0.01	ND	0.31 ± 0.04	1.42 ± 0.15	0.04 ± 0.02	0.34 ± 0.04	3.06 ± 0.37	1.25 ± 0.07	1.33 ± 0.06	0.83 ± 0.05	0.51 ± 0.02	1.04 ± 0.11	32.00 ± 0.52	1.70 ± 0.04	0.93 ± 0.10	50.66 ± 1.52
42	0.05 ± 0.00	0.04 ± 0.00	0.06 ± 0.00	0.15 ± 0.01	1.28 ± 0.05	Trace	1.52 ± 0.03	0.10 ± 0.01	ND	0.07 ± 0.01	0.99 ± 0.06	0.02 ± 0.00	0.21 ± 0.02	5.52 ± 0.22	1.29 ± 0.04	1.39 ± 0.08	0.44 ± 0.04	0.65 ± 0.03	0.28 ± 0.03	29.53 ± 0.84	1.48 ± 0.06	0.54 ± 0.03	45.59 ± 1.37
43	0.13 ± 0.01	0.09 ± 0.00	0.13 ± 0.00	0.25 ± 0.01	2.56 ± 0.08	0.02 ± 0.00	2.02 ± 0.03	0.22 ± 0.00	ND	0.23 ± 0.02	1.25 ± 0.17	0.03 ± 0.00	0.30 ± 0.02	3.24 ± 0.22	0.32 ± 0.01	0.32 ± 0.01	0.66 ± 0.10	0.47 ± 0.01	0.20 ± 0.02	27.81 ± 0.15	1.34 ± 0.07	0.79 ± 0.10	42.38 ± 1.27
44	0.07 ± 0.00	0.06 ± 0.00	0.07 ± 0.00	0.33 ± 0.00	2.06 ± 0.03	0.01 ± 0.00	1.33 ± 0.02	0.15 ± 0.01	ND	0.10 ± 0.01	0.98 ± 0.04	0.03 ± 0.01	0.14 ± 0.02	1.70 ± 0.13	0.95 ± 0.08	1.03 ± 0.05	0.42 ± 0.05	0.44 ± 0.04	0.28 ± 0.04	21.81 ± 0.84	1.02 ± 0.02	0.41 ± 0.05	33.38 ± 1.00
45	0.36 ± 0.03	0.25 ± 0.00	0.30 ± 0.01	0.53 ± 0.02	3.81 ± 0.07	0.10 ± 0.01	2.27 ± 0.12	0.38 ± 0.01	ND	0.47 ± 0.05	1.52 ± 0.07	0.03 ± 0.01	0.33 ± 0.03	3.72 ± 0.23	1.96 ± 0.09	1.99 ± 0.09	1.09 ± 0.15	0.65 ± 0.06	0.64 ± 0.08	43.07 ± 0.95	2.28 ± 0.12	0.97 ± 0.11	66.74 ± 2.00
46	0.03 ± 0.00	0.04 ± 0.00	0.05 ± 0.00	0.09 ± 0.01	1.21 ± 0.02	Trace	0.74 ± 0.04	0.07 ± 0.00	ND	0.04 ± 0.00	1.11 ± 0.06	Trace	0.13 ± 0.02	7.57 ± 0.65	0.77 ± 0.05	0.72 ± 0.09	0.68 ± 0.09	0.29 ± 0.04	0.26 ± 0.03	37.51 ± 0.60	1.66 ± 0.06	0.47 ± 0.02	53.42 ± 1.60
47	0.22 ± 0.00	0.16 ± 0.01	0.14 ± 0.00	0.50 ± 0.01	1.54 ± 0.06	0.09 ± 0.01	2.53 ± 0.08	0.34 ± 0.02	ND	0.25 ± 0.02	1.06 ± 0.14	0.06 ± 0.01	0.17 ± 0.02	10.94 ± 0.82	1.35 ± 0.06	1.48 ± 0.07	0.55 ± 0.06	0.48 ± 0.04	0.42 ± 0.05	44.83 ± 0.64	1.77 ± 0.14	0.61 ± 0.09	69.50 ± 2.09
48	0.18 ± 0.01	0.14 ± 0.00	0.17 ± 0.00	0.45 ± 0.02	3.45 ± 0.12	0.05 ± 0.01	1.96 ± 0.05	0.24 ± 0.01	ND	0.22 ± 0.03	1.54 ± 0.12	0.03 ± 0.00	0.12 ± 0.01	2.67 ± 0.05	1.50 ± 0.06	1.50 ± 0.08	0.71 ± 0.09	0.29 ± 0.03	0.95 ± 0.13	31.68 ± 0.67	1.34 ± 0.02	0.62 ± 0.02	49.81 ± 1.49
49	0.35 ± 0.03	0.20 ± 0.01	0.25 ± 0.01	0.30 ± 0.01	3.86 ± 0.29	0.07 ± 0.01	1.67 ± 0.05	0.26 ± 0.01	ND	0.25 ± 0.04	1.62 ± 0.12	0.03 ± 0.00	0.15 ± 0.02	5.41 ± 0.50	0.48 ± 0.05	0.50 ± 0.06	0.69 ± 0.08	Tarce	2.36 ± 0.25	24.86 ± 1.41	1.12 ± 0.03	0.60 ± 0.05	45.01 ± 1.35
50	0.05 ± 0.01	0.05 ± 0.00	0.08 ± 0.00	0.24 ± 0.01	3.44 ± 0.05	0.04 ± 0.01	1.23 ± 0.01	0.12 ± 0.02	ND	0.18 ± 0.02	1.95 ± 0.14	0.07 ± 0.01	0.36 ± 0.05	2.33 ± 0.26	0.17 ± 0.00	0.25 ± 0.02	0.41 ± 0.06	0.13 ± 0.01	0.24 ± 0.00	33.95 ± 0.66	1.81 ± 0.07	0.60 ± 0.07	47.69 ± 1.43
mean	0.23 ± 0.13	0.16 ± 0.08	0.22 ± 0.14	0.44 ± 0.21	2.56 ± 0.67	0.05 ± 0.03	1.88 ± 1.21	0.29 ± 0.17	-	0.25 ± 0.14	2.03 ± 1.55	0.02 ± 0.03	0.28 ± 0.17	2.33 ± 2.19	1.70 ± 1.16	1.78 ± 1.21	0.50 ± 0.20	0.52 ± 0.34	1.04 ± 1.02	41.86 ± 15.56	2.11 ± 1.13	1.54 ± 1.73	

[a] The sample No. is same as Table 3; [b] The analytes is same as in Table 1; [c] Not detected; [d] Under the LOQ.
